# The impact of positive experiences in childhood and adolescence on later mental health: A systematic review of longitudinal cohort studies

**DOI:** 10.1002/jcv2.70172

**Published:** 2026-11-13

**Authors:** Lisa H. Telford, Colm Healy, Éilis J. O'Reilly, Lama Alquthami, Amna Al Hitmi, Sami Almasri, Mary C. Clarke

**Affiliations:** ^1^ Department of Health Psychology School of Population Health Royal College of Surgeons in Ireland Dublin Ireland; ^2^ School of Medicine University College Dublin Dublin Ireland; ^3^ School of Public Health University College Cork Cork Ireland; ^4^ School of Medicine Royal College of Surgeons in Ireland Dublin Ireland; ^5^ Department of Psychiatry Royal College of Surgeons in Ireland Dublin Ireland

**Keywords:** childhood experiences, mental health, positive experiences, promotive factors

## Abstract

**Background:**

Positive experiences in early life are increasingly recognised as important contributors to mental health across the lifespan. While adverse childhood experiences have been extensively studied, the role of singular and cumulative positive experiences remains less well understood. This systematic review synthesises longitudinal evidence on associations between empirically measured positive experiences in childhood or adolescence and adult mental health outcomes.

**Methods:**

Four databases (APA PsycInfo, Embase, MEDLINE, and Scopus) were searched from inception to January 2024 and updated in May 2026. Eligible studies were longitudinal cohort studies examining associations between exposure to singular or cumulative positive experiences from birth to age 18 and adult outcomes of depression, anxiety, psychosis, or bipolar disorder, measured through clinical diagnoses, symptom‐level assessments, or broader psychopathology indices. Risk of bias was assessed using the Joanna Briggs Institute Critical Appraisal Checklist, and findings were narratively synthesised. The protocol was registered on PROSPERO (CRD42023482969).

**Results:**

20 studies (*N* = 63,271) were included. Physical activity, engaged parenting, peer social support, school belonging, school connectedness, religious service attendance, and cumulative positive experiences were associated with a reduced risk of adult depression. School belonging, school connectedness, perceived support from family and friends, and a greater number of positive relative to adverse experiences were associated with lower levels of anxiety. A positive home atmosphere, greater social cohesion, higher levels of physical and leisure‐time activity, and participation in sports competitions were linked to fewer psychotic symptoms. No studies examined bipolar disorder. All studies were of moderate to high quality, although definitions and measurement of positive experiences varied.

**Conclusion:**

Positive experiences in childhood and adolescence may contribute to better mental health outcomes in adulthood. However, further longitudinal research is needed to clarify mechanisms, timing, and cumulative effects, and to standardise measurement across diverse populations.

## Key Points

**What's known?**

- Positive experiences in childhood are increasingly being recognised as important contributors to later mental health.

**What's new?**

- Longitudinal evidence on positive experiences remains limited and conceptually fragmented.

**What's relevant?**

- Mixed findings indicate benefit from several singular as well as cumulative positive experiences on later mental health, which may inform early intervention and policy initiatives.
- Researchers should include standardised cumulative positive experience measures within longitudinal studies to facilitate future cross‐cohort comparisons.

## INTRODUCTION

Mental disorders are the leading cause of disability among young people worldwide, accounting for a quarter of years lost due to disability between ages 0 and 24 (Erskine et al., 2015). Risk for mental illness peaks in adolescence and young adulthood, with most disorders emerging by the age of 25 (McGorry et al., 2024). While the developmental origins of mental illness are complex and multifactorial (Tsuang et al., 2004), early life environments, both helpful and harmful, provide the bedrock for development over the life course and play a critical role in shaping long‐term outcomes (Cicchetti & Toth, 2009; Masten, 2006).

Research on adverse childhood experiences (ACEs), such as abuse, neglect, and household dysfunction, has consistently demonstrated their detrimental impact on adult mental and physical health (Abbott & Slack, 2021; Felitti et al., 1998). However, growing attention is being paid to the role of positive experiences in early life, which may promote resilience and mitigate the effects of adversity. Crucially, these experiences are not merely the absence of adversity but rather additional experiences, resources and assets that foster healthy development and psychosocial well‐being (Daines et al., 2021; Han et al., 2023). Although terminology varies (Raghunathan et al., 2024), positive experiences are typically conceptualised within four domains: supportive and nurturing relationships; safe, stable and protective environments; opportunities for social engagement and connectedness; and social and emotional development (Narayan et al., 2018; Sege & Harper Browne, 2017).

Despite increasing interest, the literature on positive experiences remains conceptually fragmented. Some studies retrospectively assess cumulative exposure using validated scales, while others examine singular experiences such as parental involvement, peer support, or school connectedness. Importantly, positive experiences may exert promotive effects, improving outcomes regardless of adversity exposure, or protective effects, buffering against the negative impact of adversity (Brumley & Jaffee, 2016; Han et al., 2023; Samji et al., 2025). However, these distinctions are not always clearly operationalised, and evidence from longitudinal studies, the design features of which are critical for exploring temporality, supporting causal inference and classifying developmental trajectories with a view to identifying opportunities for intervention remains limited (Bogdan et al., 2023).

Previous reviews have either focussed on cross‐sectional data (Han et al., 2023), explored psychosocial outcomes broadly (Fritz et al., 2018), or examined resilience factors within adversity‐exposed samples (Buchanan et al., 2023), limiting the generalisability of findings. To date, few reviews have synthesised longitudinal evidence (Kallapiran et al., 2025) and none have specifically investigated prospective associations between positive experiences in childhood, whether singular or cumulative, and adult mental health outcomes in general population samples.

This systematic review aims to address this gap by identifying and summarising evidence from longitudinal cohort studies that examine the relationship between positive experiences from birth to age 18 and the development of depression, anxiety, psychosis, or bipolar disorder or symptoms of these disorders in adulthood. By integrating findings from published observational studies, we seek to clarify the promotive potential of positive experiences in shaping long‐term mental health outcomes in the general population.

## METHODS

This systematic review was conducted in accordance with COSMOS‐E guidelines for reviews of observational studies (Dekkers et al., 2019) and reported following the PRISMA 2020 Statement (Page et al., 2021). Details of this review can be found in the PRISMA checklists (L. Telford, 2025a, 2025b).

### Protocol and registration

The protocol was registered with PROSPERO under CRD42023482969 (https://www.crd.york.ac.uk/PROSPERO/view/CRD42023482969) and published on Health Research Board (HRB) Open Research (L. H. Telford et al., 2024).

### Eligibility criteria

Studies were eligible if they met the following criteria: (a) peer‐reviewed empirical longitudinal cohort studies in humans; (b) published in English; (c) assessed exposure to singular positive experiences or cumulative indices of such experiences occurring between birth and age 18; and (d) examined associations with adult mental health outcomes, including clinically diagnosed depression, anxiety, psychotic, or bipolar disorders, symptom‐based measures of these disorders, or multidimensional measures of psychopathology in adulthood (age >18). Studies were excluded if they: (a) did not report original data (e.g., reviews, editorials, conference abstracts); (b) used selected or high‐risk samples (e.g., clinical or trauma‐exposed cohorts), to maintain generalisability; (c) assessed exposure retrospectively in adulthood; (d) measured outcomes in childhood; or (e) reported lifetime diagnoses without age‐specific data. See Supporting Information S1: Table S1 for the detailed list of criteria.

For review purposes, childhood was defined as birth to age 18 and adulthood as 18 years and above. Studies that assessed either singular or cumulative positive experiences in childhood were eligible. This approach was adopted to reflect the heterogeneity of the longitudinal literature, where many studies examine discrete experiential domains without constructing composite exposure measures. Studies assessing singular positive experiences were included, provided the exposure(s) aligned conceptually with our operational definition.

Positive experiences were operationally defined as those occurring within safe, stable, and nurturing relationships and environments which support healthy development and promote resilience, consistent with the Health Outcomes from Positive Experiences (HOPE) framework (Sege & Harper Browne, 2017). We distinguished exposure to positive experiences from (a) the absence of adversity, (b) neurocognitive traits (e.g., IQ), (c) individual behaviours lacking relational or environmental context (e.g., religious affiliation), and (d) attachment security and general parenting constructs (e.g., parenting style typologies) unless explicitly framed as a discrete positive experiential exposure. To ensure conceptual alignment, spirituality‐related exposures were only eligible where they reflected experiential or participatory dimensions such as the subjective importance of faith or involvement in communal religious or spiritual activities. We explicitly chose to only include evidence from longitudinal studies where the exposure was measured in childhood, as the assessment of past exposure (i.e., recollecting experiences in childhood or adolescence as an adult) is subject to recall bias and more likely to result in subjective measures (Colman et al., 2016).

We focussed on anxiety, major depressive, bipolar and psychotic disorder, as depressive and anxiety disorders are the most prevalent and therefore have the greatest potential for impact through targeted preventive and therapeutic intervention (GBD, 2019 Mental Disorders Collaborators, 2022), while psychosis and bipolar disorder have significant detrimental effects on lives due to their chronic nature, difficult treatment and often early onset (Robinson & Bergen, 2021). We chose to include studies reporting symptoms of our outcomes of interest as well as general adult psychopathology to capture subthreshold cases, since the prevalence of clinically diagnosed mental illness is known to be relatively low in population‐based samples (Demyttenaere et al., 2004).

### Information sources

Four electronic databases; APA PsycInfo (EBSCO platform), Embase (Elsevier platform), MEDLINE (Ovid platform), and Scopus were systematically searched from inception until January 2024 to identify relevant studies. In addition, published reviews in the field, the reference lists of included studies, and relevant grey literature were manually searched to identify further eligible studies.

### Search strategy

The search strategy was designed to identify relevant longitudinal studies examining associations between singular or cumulative positive experiences and adult mental health outcomes. Singular positive experiences were operationalised according to the most widely used scale at the time of searching, the Benevolent Childhood Experiences (BCEs) scale (Narayan et al., 2018). The 10 BCEs were supplemented with eight additional experiences drawn from early foundational literature and related resilience measures (Abbott & Slack, 2021; Hays‐Grudo & Morris, 2020; Ungar & Liebenberg, 2011) to capture relational and environmental experiential exposures consistent with the HOPE framework (Sege & Harper Browne, 2017).

In total, 18 positive experience terms were included in the search strategy (one safe caregiver, friends, comforting beliefs, school enjoyment, a caring teacher, good neighbours, adult mentor, opportunities, positive self‐image, home routines, community traditions, spiritual beliefs, education, school belonging, skills development, household safety, physical activity, and hobbies) alongside known alternate terms for cumulative positive experiences (i.e., counter ACEs, advantageous, benevolent, protective and compensatory childhood experiences). These exposure terms were selected based on the best available conceptual and empirical literature at the time of searching in order to provide a focussed collation of the evidence base.

All four database searches were conducted on 26 January 2024 and results stored in separate text files. No limits were placed on publication date; however, searches were restricted to articles published in English. An updated search was conducted on 20 May 2026, with newly retrieved records screened using a rapid screening approach. At the same time, targeted hand‐searching of relevant sources was undertaken to identify additional eligible studies. See Supporting Information S1: Table S2 for the final database‐specific search strategies.

### Selection process

Citations were imported directly into Covidence (https://www.covidence.org/), an online collaborative software tool designed to simplify the systematic review process (Covidence systematic review software, n.d.). Deduplication was automatically carried out by the software, and any further duplicates were identified manually during the pre‐screening process. Inclusion and exclusion criteria were applied to titles and abstracts to establish eligibility, followed by full‐text. Studies identified through manual hand‐searching were screened for eligibility in the same manner. The first author conducted the search and screened all records independently. Three further reviewers independently screened all records, and full‐text screening conflicts were resolved through discussion with an independent, third‐party reviewer. Microsoft Copilot (accessed June 2026) was used to assist with the rapid screening of potentially eligible studies identified during the updated search by applying predefined inclusion/exclusion criteria.

### Data extraction and quality assessment

A tailor‐made data extraction form was created and piloted. The first author then extracted predefined data items relating to the study, population and setting, exposure(s), mental health outcome(s) and measures of effect from each included study. Corresponding authors were emailed when articles lacked relevant information or reported incongruent metrics. Only data pertaining to relevant analyses were extracted from included studies. See Supporting Information S1: Table S3 for the full list of data extraction items.

The quality of included studies was assessed by the first author using the Joanna Briggs Institute (JBI) Critical Appraisal Checklist for Cohort Studies (Barker et al., 2023). An overview of the methodological quality of included studies is presented graphically and key findings are reported narratively.

### Data synthesis

Given the absence of common model parameters for the outcomes of interest among included studies, a meta‐analysis was not performed. Instead, a narrative synthesis was conducted. To conceptually organise the exposures from included studies, we mapped them onto Bronfenbrenner's bioecological model (Bronfenbrenner & Morris, 2006). While the model conceptualises development as arising from dynamic interactions across systems (Lopez et al., 2021), it was used here as an organisational heuristic to situate singular positive experience exposures within broader ecological contexts, rather than to imply independent or causal effects. Only the final models for relevant exposure/outcome associations, adjusted for covariates, are discussed. Characteristics of included studies are presented graphically, their relevant results summarised and key findings reported by bioecological domain. As data were insufficient for subgroup analysis, we have reported exposure measurement type in Table 1 to capture frequency.

**TABLE 1:** Summary of characteristics of included studies (*N* = 20).

First author & year	Setting, analytic sample (*n*) & % female	Cohort	Exposure(s)	Exposure measure & age at assessment	Outcome(s)	Outcome measure^a^ & age at assessment	Metrics, analysis method & adjustments
Allen et al. (2024)	Australia, *n* = 1568 (51% female)	ATP	School belonging	Self‐report via the short form of the ACER school life questionnaire at 15–16 years	Symptoms of depression and anxiety	Self‐report via the short‐form DASS‐21^a^ at 19–20, 23–24 and 27–28 years	*β* and 95% CI using GEEs adjusted for parent country of birth, parental separation/divorce, low parental education, participant sex at birth, prior internalising and externalising problems, antisocial behaviour across ages 13–18 years
Beyers and Seiffge‐Krenke (2007)	Germany, *n* = 228 (52% female)	Prospective longitudinal study	Social support in relationships and changes in family relationships	Adolescent‐report via the German version of the NRI at 17 years (*social support*) and adolescent‐, mother‐ and father‐report via the FES at 14–17 years (*family relationships*)	Levels of psychopathology	Self‐report via the SCL‐90‐R^a^ at 21 years	Std. *β* and *p*‐value using mediation analysis adjusted for family relationship trajectory classes and stratified by sex
Brunet et al. (2013)	Canada, *n* = 816 (53% female)	NDIT study	Moderate to vigorous physical activity and involvement in organised team sports	Self‐report via repeated in‐school questionnaires throughout secondary school (from 13 to 14 until 17–18 years)	Depressive symptoms	Self‐report via the MDI^a^ at 18–24 years	Std. *β* and 95% CI using multiple hierarchical linear regression adjusted for self‐reported age, sex, depressive symptoms at baseline and parental university education
Cong et al. (2020)	UK, *n* = 7120 (49% female)	ALSPAC	Parental involvement	Parent‐report via questionnaire on seven occasions during the offspring's childhood	Depression	Self‐report via a computerised version of the CIS‐R^a^ at 18 years	OR and 95% CI using logistic regression adjusted for parental marital status, maternal age at delivery, maternal smoking status, maternal mental health, paternal smoking status, and paternal mental health
Crandall et al. (2020)	USA, *n* = 489 (51% female)	FFP	Ten counter‐ACEs and eight ACEs	Adolescent‐ and parent‐report via surveys	Depressive and anxiety symptoms	Self‐report via the CES‐DC^a^ and generalised anxiety disorder subscale of the SCAS^a^ at 19–22 years	Std. *β* and *p*‐value using SEM adjusted for adolescent gender, adolescent age at baseline, adolescent race, and sampling method
Crush et al. (2018)	UK, *n* = 2063 (sex not specified)	E‐risk longitudinal twin study	Home atmosphere and social cohesion	Interviewer‐rated post study visit via the CII at 12 years (*atmosphere at home*) and estimated via postal survey of residents living alongside E‐risk families at 13/14 years (*social cohesion*)	Clinically‐verified psychotic symptoms	Clinically verified via private interview at 18 years	Unadjusted OR and 95% CI using logistic regression due to the small number of individuals with psychotic symptoms
da Silva et al. (2023)	Brazil, *n* = 3247 (53% female)	1993 Pelotas (Brazil) birth cohort study	Leisure‐time physical activity and total physical activity (leisure plus commuting)	Self‐report via questionnaire at 11 and 15 years	Major depressive disorder and generalised anxiety disorder	Clinical diagnosis by trained psychologists using the MINI at 22 years	PR and 95% CI using Poisson regression with robust error variance adjusted for sex, family income at birth, maternal schooling at birth, skin colour, BMI at 11 and 15 years, screen time at 11 and 15 years, and total SDQ score at 11 and 15 years
Glickman et al. (2021)	UK, *n* = 3263 (64% female)	ALSPAC	Perceived peer social support and childhood emotional neglect (both included as independent predictors in multivariable models)	Self‐report via the 5‐item cambridge hormones and moods Project friendship questionnaire at 15 years (*peer social support*) and self‐report via eight separate assessments from 8 to 17.5 years of age (*childhood emotional neglect*)	Depressive symptoms	Self‐report via the SMFQ^a^ at 18 years	β and 95% CI using linear regression adjusted for the child's sex, child's race, mother's age at child's birth, number of previous pregnancies, marital status, home ownership status, and mother's educational attainment
Gondek et al. (2023)	UK, *n* = 4490 (58% female)	ALSPAC	Total positive experience count with a range of 0–11	Self‐report by study children or their parents when study children were between 9 and 14 years of age	Depressive symptoms	Self‐report via the SMFQ^a^ at 18 years	Poisson beta coefficient and 95% CI using GLM with a log link function and robust standard errors adjusted for child's sex, family's ethnicity, maternal partnership status, parental education, parental social class, financial difficulties, housing tenure, crowding index, mother smoking during pregnancy, mother drinking alcohol during pregnancy, mother's age at birth, parental mental health problems
Howie et al. (2020)	Australia, *n* = 1628 (48% female)	Raine study—Generation 2 (Gen2)	Physical activity trajectories	Constructed using latent class analysis from parent‐report of relative activity via questionnaire at 8, 10, 14, and 17 years	Depression and anxiety symptoms	Self‐report via the DASS‐21^a^ short form at 22 years	Mean and 95% CI using negative binomial regression stratified by sex
Kandola et al. (2020)	UK, *n* = 4257 (56% female)	ALSPAC	Physical activity	Objectively measured via uniaxial accelerometer at 12, 14 and 16 years	Depressive symptoms	Self‐report via a computerised version of the CIS‐R^a^ at 18 years	IRR and 95% CI using separate negative binomial regression models adjusted for sex, maternal social class, parental psychiatric history, parental education, ethnicity, baseline depression, and total accelerometer wear time at each time point
Kemp et al. (2024)	Australia, *n* = 3708 (50% female)	LSAY	Cumulative PYE score with a range of 0–10	Self‐report via annual survey between 15 and 17 years of age	Mental health	Self‐report via the 6‐item short form K6^a^ at 25 years	OR and 95% CI using multivariate logistic regression adjusted for school sector, sex, indigenous status, rurality, highest parental income, and exposure to AYEs
Låftman et al. (2025)	Sweden, *n* = 2722 (58% female)	Futura01	Perceived social support (family, friend and significant other)	Self‐report via the MSPSS at 17–18 years	Depression and anxiety symptoms	Self‐report via the PHQ‐4^a^ at 20–21 years	OR and 95% CI using logistic regression including all sources of perceived social support adjusted for gender, other sociodemographic characteristics, and prior mental health problems
McKercher et al. (2014)	Australia, *n* = 1630 (53% female)	The childhood determinants of adult health study (2004–2006)	Discretionary (leisure) physical activity	Self‐report via supervised questionnaire at 9–15 years	Depression (major depressive or dysthymic disorder)	Clinical diagnosis based on DSM‐IV criteria via participant completion of the depression module of the self‐administered computerised CIDI‐Auto^a^, version 2.1 at 27–36 years	RR and 95% CI using log‐binomial regression adjusted for mother's place of birth, childhood smoking status, age in adulthood and stratified by sex
Nyberg et al. (2019)	Sweden, *n* = 1083 (47% female)	Northern Swedish cohort	School connectedness and family climate	Self‐report via questionnaire at 16 years	Symptoms of depression and anxiety	Self‐report via questionnaire (six depression and five anxiety items) at 43 years	β and 95% CI using SEM adjusted for gender, symptoms of depression at baseline, mother's and father's socio‐economic status, and mother's and father's mental and physical health
Rollins and Crandall (2021)	USA, *n* = 489 (51% female)	FFP	Childhood experiences (eight ACEs and 10 PCEs)	Adolescent‐ and parent‐report via surveys	Mental health indicators, including depression and anxiety	Self‐report via the 20‐item CES‐DC^a^ and the 6‐item SCAS^a^ at 20–23 years	Correlation coefficients and *p*‐values using pairwise correlation matrix
Sagatun et al. (2007)	Norway, *n* = 2433 (55% female)	Oslo Health study	Physical activity	Self‐report via questionnaire at 15–16 years	Mental health (emotional and behavioural difficulties)	Self‐report via the SDQ^a^ with impact supplement at 18–19 years	Adjusted mean with 95% CI using multiple linear regression adjusted for invitation group, ethnicity, family economy, smoking, use of alcohol, respective SDQ subscore at baseline and stratified by sex
Sormunen et al. (2017)	Finland, *n* = 3596 (sex not specified)	YFS	Physical activity	Self‐report via questionnaire at 9, 12, 15, and 18 years	Hospital‐related psychiatric diagnoses, including non‐affective psychosis and schizophrenia	Clinical diagnosis based on DSM‐IV criteria converted from ICD diagnoses via the Finnish national hospital discharge register	RR with 95% CI using multivariable modified Poisson regression adjusted for sex, age, BMI in childhood and adolescence, birth weight, non‐preterm birth, and mother's mental disorders
Steiner et al. (2019)	USA, *n* = 14,800 (49% female)	National longitudinal study of adolescent to adult health (add health)	School and family connectedness	Self‐report via in‐school questionnaires and in‐home student and parent interviews at 12/13 to 17/18 years	Emotional distress	Self‐report via a 10‐item CES‐D‐derived depressive symptom scale (add health wave 4 short form) at 24–32 years	β and SE using multivariable linear regression including both school and family connectedness adjusted for age, sex, race and ethnicity, parent's highest level of education, parent's marital status, receipt of public assistance, and baseline emotional distress
Upenieks and Thomas (2021)	USA, *n* = 3641 (52% female)	NLSY79	Religious service attendance	Self‐report via interview at 14–18 years	Depressive symptoms	Self‐report via a nine‐item version of the CES‐D^a^ at 50–54 years	Unstandardised *β* and SE using OLS regression adjusted for gender, race, age (in 2014), parental education (head of household), family structure, and being raised in the american south

Abbreviations: ACER, Australian Council for Educational Research; β, Beta/regression coefficient; ACEs, adverse childhood experiences; ALSPAC, Avon Longitudinal Study of Parents and Children; ATP, Australian Temperament Project; AYE, adverse youth experience; BMI, body mass index; CES‐D, Centre for Epidemiologic Studies Depression Scale; CES‐DC, Centre for Epidemiological Studies Depression Scale for Children; CI, confidence interval; CIDI, Composite International Diagnostic Interview; CII, Coder's Impression Inventory; CIS‐R, Clinical Interview Schedule‐Revised; DASS‐21, Depression Anxiety and Stress Scales; DSM‐IV, Diagnostic and Statistical Manual of Mental Disorders, Fourth Edition; E‐Risk, Environmental Risk; FES, Family Environment Scale; FFP, Flourishing Families Project; GEEs, generalised estimating equations; GLM, generalised linear model; ICD, International Classification of Diseases; IRR, incidence rate ratio; K6, Kessler Psychological Distress measure; LSAY, Longitudinal Surveys of Australian Youth; MDI, Major Depression Inventory; MINI, Mini International Neuropsychiatric Interview; MSPSS, Multidimensional Scale of Perceived Social Support; NDIT, Nicotine Dependence in Teens; NLSY79, National Longitudinal Study of Youth; NRI, Network of Relationships Inventory; OLS, ordinary least squares; OR, odds ratio; PCEs, positive childhood experiences; PHQ‐4, Patient Health Questionnaire‐4; PR, prevalence ratio; PYE, positive youth experience; RR, relative risk/risk ratio; SCAS, Spence Children's Anxiety Scale; SCL‐90‐R, Symptom Checklist 90‐R; SDQ, Strengths and Difficulties Questionnaire; SE, standard error; SEM, structural equation modelling; SMFQ, Short Mood and Feelings Questionnaire; Std. *β*, standardised regression coefficient; UK, United Kingdom; USA, United States of America; YFS, Cardiovascular Risk in Young Finns.

^a^ Validated instrument.

## RESULTS

### Study selection

The search yielded 4397 records in total, 1265 of which were duplicates and were removed. A total of 3132 records were duplicate screened on title and abstract by independent reviewers. Of these, 3099 were deemed ineligible, and 33 records were retrieved for full‐text review. Twenty‐one studies were excluded on full‐text review, with the most frequent reasons for exclusion being the use of selected and/or high‐risk samples, measurement of the outcome in childhood (i.e., before age 18), and ineligible study type (e.g., abstract only). Thirty‐five additional records were identified via hand‐searching, all of which were screened on full‐text for eligibility. Of these, four were deemed eligible for inclusion.

An updated search conducted in May 2026 identified 1211 additional records, from which one study met the inclusion criteria following rapid screening. Concurrent targeted hand‐searching identified a further three eligible studies. A total of 20 studies (*N* = 63,271) met our eligibility criteria and have been included in this review. The PRISMA flow diagram (Figure 1) reflects the study selection process and reasons for exclusion based on full‐text review for the original search conducted on 26 January 2024 (Page et al., 2021). The full list of studies excluded at the full‐text screening stage with detailed reasons for exclusion can be found in Supporting Information S1: Table S4.

**FIGURE 1:**
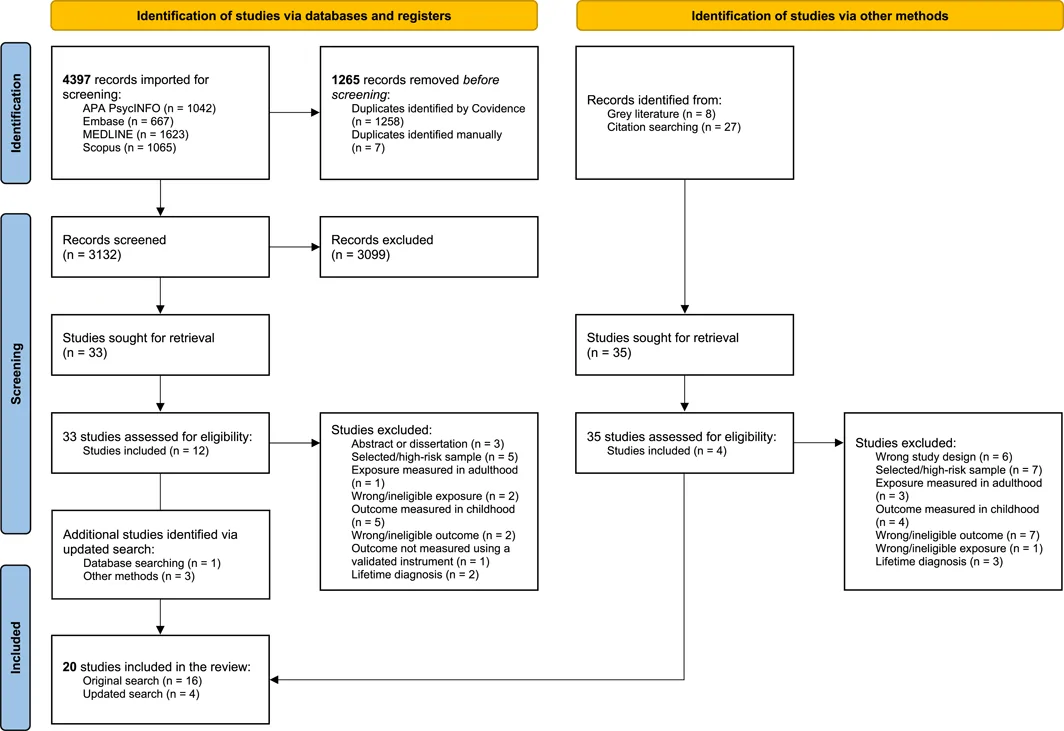
PRISMA flow diagram. The final inclusion count incorporates four additional studies identified through an updated search conducted in May 2026. As records from the original and updated searches were merged within Covidence, the original screening pathway has been retained.

### Quality of included studies

The JBI Critical Appraisal Checklist for Cohort Studies (Barker et al., 2023) was used to rate the methodological quality of each included study (see Supporting Information S1: Table S5). Total possible scores ranged from 0% to 100%, with a higher percentage indicating better quality. Percent scores were calculated by summing the number of checklist items marked ‘yes’ divided by the total possible of 11 for each included study. For ease of interpretation, we categorised quality scores into three groups: 0%–36% indicating low, 46%–73% moderate, and 82%–100% high quality.

The methodological quality of the majority of studies was high (*n* = 14), with only one scoring a maximum of 100%. The quality of the remaining four studies was deemed moderate (*n* = 6), and none were found to be of low quality. Overall, sample attrition, reasons for loss to follow‐up and strategies to address incomplete follow‐up were poorly reported among included studies. Similarly, several studies did not explicitly report whether or not participants were free of the outcome at the outset of the study, and exposure assessment was unclear among several included studies.

### Sample characteristics

All included studies utilised datasets from prospective longitudinal cohorts. The median follow‐up duration was 10 years, with the shortest being 3 years (Låftman et al., 2025; Sagatun et al., 2007) and the longest over 35 years (Upenieks & Thomas, 2021). Sample sizes varied in range from 228 participants (Beyers & Seiffge‐Krenke, 2007) in the smallest to over 14,000 (Steiner et al., 2019) in the largest study. Most studies were published within the past 5 years, and all within the last 20 years. All studies, with one exception, were conducted in high‐income countries. Of the 20 included studies, five (Cong et al., 2020; Crush et al., 2018; Glickman et al., 2021; Gondek et al., 2023; Kandola et al., 2020) used data from United Kingdom participants, four (Allen et al., 2024; Howie et al., 2020; Kemp et al., 2024; McKercher et al., 2014) used data from Australian cohorts, four (Crandall et al., 2020; Rollins & Crandall, 2021; Steiner et al., 2019; Upenieks & Thomas, 2021) were conducted using American samples, two (Låftman et al., 2025; Nyberg et al., 2019) used Swedish data, and the remainder were spread equally among Brazil (da Silva et al., 2023), Canada (Brunet et al., 2013), Finland (Sormunen et al., 2017), Germany (Beyers & Seiffge‐Krenke, 2007), and Norway (Sagatun et al., 2007) (one each). The majority of included studies (*n* = 11) had a near‐equal split in sex at 49%–53% female. Five studies had a higher female predominance (55%–64%), peaking at 64% in one study (Glickman et al., 2021). The proportions of participants by sex were not reported in two studies (Crush et al., 2018; Sormunen et al., 2017). Participant age at enrolment ranged from 4–8 months (Allen et al., 2024) to 16 years (Nyberg et al., 2019), while seven studies were birth cohorts (Cong et al., 2020; Crush et al., 2018; da Silva et al., 2023; Glickman et al., 2021; Gondek et al., 2023; Howie et al., 2020; Kandola et al., 2020).

Sixteen studies (Allen et al., 2024; Beyers & Seiffge‐Krenke, 2007; Brunet et al., 2013; Crandall et al., 2020; da Silva et al., 2023; Glickman et al., 2021; Gondek et al., 2023; Kemp et al., 2024; Låftman et al., 2025; McKercher et al., 2014; Nyberg et al., 2019; Rollins & Crandall, 2021; Sagatun et al., 2007; Sormunen et al., 2017; Steiner et al., 2019; Upenieks & Thomas, 2021) investigated one or more exposures measured via self‐report in childhood and/or adolescence. Six studies (Beyers & Seiffge‐Krenke, 2007; Cong et al., 2020; Crandall et al., 2020; Howie et al., 2020; Rollins & Crandall, 2021; Steiner et al., 2019) explored one or more exposures measured via parent‐ or family‐report in childhood and/or adolescence. Exposure(s) were objectively measured in childhood and adolescence in one study (Kandola et al., 2020), interviewer‐rated in childhood in another (Crush et al., 2018) and estimated via survey in adolescence in the same study (Crush et al., 2018). Characteristics of included studies are reported in Table 1.

### Types of positive experiences

Studies included in this review examined associations between singular or cumulative positive experiences and adult mental health outcomes across three categories; anxiety, depression, and psychosis, alongside broader indices of adult psychopathology. In line with recommendations by Lopez et al. (2021), identified exposures have been mapped onto Bronfenbrenner's bioecological model (Bronfenbrenner & Morris, 2006). Using a social ecology lens to organise the findings of this narrative synthesis, we hope to demonstrate the breadth of positive experiences and build towards a holistic understanding of the interrelated impact of various early life experiences on adult mental health. Exposures from included studies fell within the domains of the individual, family/household, friends/peers, school, and community, all of which are situated at the individual or microsystem levels. Studies which investigated multiple positive experiences using a cumulative approach are discussed separately.

#### Individual

Seven of the 20 included studies investigated positive experiences within the individual domain. All seven explored associations between physical activity, taken as an indicator of participation in socially embedded activities, and mental health outcomes. Evidence from four high‐quality studies and one moderate‐quality study found mixed results for depression. da Silva et al. (2023) found that participants meeting World Health Organisation guidelines for physical activity in leisure time at 11 years old (at least 300 minutes per week) were 46% less likely to receive a diagnosis of major depressive disorder at 22 years (PR = 0.54, 95% CI: 0.33–0.89), while participants that met recommendations for total physical activity (leisure time plus commuting) at 11 years old were 37% less likely to receive a diagnosis of major depressive disorder at age 22 (PR = 0.63, 95% CI: 0.41–0.97) (da Silva et al., 2023). Howie et al. (2020) found that Depression Anxiety and Stress Scales ,DASS‐21 depression scores were lowest in the high‐activity compared to both the low‐ and mid‐activity trajectories among males, however, there were no differences in females (Howie et al., 2020).

Kandola et al. (2020) found that higher total physical activity at ages 12 and 14 years, but not at age 16 years, was associated with a lower depression score at age 18 (Kandola et al., 2020). Depression scores at 18 years were lower for every additional 60 min/day of light activity at all time points: 9.6% at 12 years, 7.8% at 14 years, and 11.1% at 16 years of age (Kandola et al., 2020). For every 15‐min increase in moderate‐to‐vigorous physical activity at age 12, but not at ages 14 or 16, depression scores at age 18 were 9.0% lower (Kandola et al., 2020). Participants with persistently high levels of light and moderate‐to‐vigorous activity had 19.6% and 31.1% lower depression scores respectively compared to those with persistently low levels (Kandola et al., 2020).

McKercher et al. (2014) found that in females, discretionary physical activity during childhood was not significantly associated with depression in adulthood (*p* > 0.05); however, a non‐significant trend among males suggested that those in the highest tertile of physical activity had approximately 55% lower risk of developing depression in adulthood compared to those in the lowest tertile (*p* = 0.06) (McKercher et al., 2014). Moderate‐quality evidence from Brunet et al. (2013) found no statistically significant associations between moderate to vigorous physical activity in secondary school and depressive symptoms in young adulthood, and a non‐significant trend towards involvement in team sports being negatively related to depressive symptoms (*p* = 0.08) (Brunet et al., 2013).

Four studies provided evidence for outcomes other than depression. Of those, two found no evidence of an association between physical activity and anxiety; da Silva et al. (2023) found that physical activity during adolescence was not associated with anxiety disorder at 22 years (da Silva et al., 2023), and Howie et al. (2020) found that there were no differences in DASS‐21 anxiety or stress sub‐scales (Howie et al., 2020).

One study by Sagatun et al. (2007) examined mental health using the Strengths and Difficulties Questionnaire and found mixed results. In boys, weekly hours of physical activity at age 15–16 years were inversely associated with emotional symptoms and peer problems at age 18–19 years but there were no statistically significant associations observed in girls (Sagatun et al., 2007). In boys, prosocial behaviour varied with physical activity but there was no linear trend while there was no independent effect of physical activity at age 15–16 on mental health at age 18–19 in girls (Sagatun et al., 2007). No statistically significant associations between physical activity at age 15–16 and conduct problems or hyperactivity‐inattention at 18–19 years were observed for neither boys nor girls (Sagatun et al., 2007).

Moderate‐quality evidence from Sormunen et al. (2017) found that low physical activity during childhood and adolescence (ages 9–18) was an independent predictor of the later development of non‐affective psychosis; a lower physical activity index (RR = 1.26, 95% CI: 1.1–1.5), reduced engagement in common leisure‐time activities (RR = 1.71, 95% CI: 1.2–2.5), and lack of participation in sports competitions (RR = 2.58, 95% CI: 1.3–5.3) were all associated with an increased risk of developing non‐affective psychosis (Sormunen et al., 2017). These findings suggest that higher levels of physical activity, greater engagement in leisure‐time activities, and participation in sports competitions during youth may be associated with a reduced risk of developing non‐affective psychosis. The same study found that physical activity level was lower among children and adolescents who later developed schizophrenia which suggests that higher levels of physical activity during youth may be associated with a lower risk of later schizophrenia (Sormunen et al., 2017). Overall, studies investigating positive experiences within the individual domain found mixed results with the most evidence for physical activity being associated with a reduced risk of depression.

#### Family/household

Four studies examined positive experiences falling within the family/household domain. Cong et al. (2020) found a one‐unit increase in overall parenting score, reflecting the total level of parental involvement in childhood, corresponded to 12% lower odds of developing depression (adjusted OR = 0.88, 95% CI: 0.79–0.98) while participants with higher average parenting scores over time had 30%–40% lower odds of developing depression in early adulthood compared to those with the lowest average parenting scores (Cong et al., 2020). Crush et al. (2018) showed that a positive atmosphere at home (OR = 0.95, 95% CI: 0.92–0.99, *p* < 0.05) was significantly associated with a reduced likelihood of psychotic symptoms at age 18 (Crush et al., 2018). Steiner et al. (2019) reported that higher family connectedness during adolescence was independently associated with lower emotional distress in adulthood (*β* = −0.07, SE = 0.02, *p* < 0.001) (Steiner et al., 2019). In a study using a multidimensional measure of perceived social support, Låftman et al. (2025) found that perceived family support was inversely associated with symptoms of both depression (OR = 0.89, 95% CI: 0.83–0.96) and anxiety (OR = 0.81, 95% CI: 0.75–0.86) in fully adjusted models (Låftman et al., 2025). Overall, evidence suggests that more engaged and involved parenting, perceived family support, a positive home atmosphere, and stronger family connectedness are associated with reduced risk of depression, anxiety, and psychotic symptoms, and lower levels of emotional distress.

#### Friends/peers

Three studies explored positive experiences within the domain of friends/peers. Beyers and Seiffge‐Krenke (2007) found that experiences of social support predicted lower levels of global psychopathology symptoms, measured using the SCL‐90, 4 years later, at age 21 (Boys *β* = −0.18, Girls *β* = −0.15) after controlling for family relationship trajectory classes (Beyers & Seiffge‐Krenke, 2007). Glickman et al. (2021) reported a significant negative association between perceived peer social support and depressive symptoms (*β* = −0.05, *p* < 0.01), indicating that as perceived peer social support increased, depressive symptoms decreased, even after adjusting for childhood emotional neglect (Glickman et al., 2021). Låftman et al. (2025) found that perceived support from friends was inversely associated with later symptoms of anxiety (OR = 0.89, 95% CI: 0.82–0.98), although no association was observed for depression (Låftman et al., 2025). Overall, evidence suggests that social support from friends and peers is associated with lower levels of psychopathology and reduced risk of anxiety and depressive symptoms, even in the context of additional stressors.

#### School

Three studies looked at positive experiences within the school domain. The study by Allen et al. (2024) found that higher levels of all aspects of school belonging were associated with lower levels of depression (overall school belonging *β* = −0.16) and anxiety (overall school belonging *β* = −0.10) following adjustments (Allen et al., 2024). Nyberg et al. (2019) found the standardised estimate for the direct path from school connectedness at age 16 to depression in adulthood to be −0.147 (*p* = 0.000) (Nyberg et al., 2019). The same study found the standardised estimate for the direct path from school connectedness at age 16 to anxiety at age 43 to also be −0.147 (*p* = 0.000) indicating that school connectedness predicts mental health symptoms in middle age (Nyberg et al., 2019). Steiner et al. (2019) found that higher school connectedness during adolescence was independently associated with lower emotional distress in adulthood (*β* = −0.06, SE = 0.02, *p* < 0.001) (Steiner et al., 2019). Overall, evidence suggests that higher levels of school belonging and stronger school connectedness are associated with lower levels of depression, anxiety, and emotional distress.

#### Community

Two studies investigated community‐domain positive experiences. Crush et al. (2018) found that higher levels of social cohesion (OR = 0.58, 95% CI: 0.34–0.99, *p* < 0.05) were statistically significantly associated with a reduced likelihood of psychotic symptoms at age 18 (Crush et al., 2018). Upenieks and Thomas (2021) found that those who were low/no religious attenders in early life had higher depression scores in later life relative to high attenders (*β* = 0.06, *p* < 0.05) which suggests that higher levels of religious service attendance in early life is associated with fewer depressive symptoms in later life (Upenieks & Thomas, 2021). Overall, evidence suggests that community‐level factors, such as social cohesion and religious service attendance, are associated with reduced risk of later mental health problems.

#### Cumulative positive experiences

Four studies examined positive experiences cumulatively, two of which used data from the same cohort, the Flourishing Families Project. Both found evidence of a negative association between cumulative positive experiences and depression and anxiety. Crandall et al. (2020) found that positive childhood experiences ,PCEs remained predictive of reduced depression (*β* = −0.11, *p* < 0.05), but were not predictive of reduced anxiety (*β* = −0.10, *p* < 0.10) after accounting for ACEs (Crandall et al., 2020). The same study also reported that as the difference score increased, indicating more positive relative to ACEs, participants reported less depression (*β* = −0.17, *p* < 0.01) and anxiety (*β* = −0.16, *p* < 0.01) (Crandall et al., 2020). In the second study, Rollins and Crandall (2021) found that PCEs were weakly negatively correlated with both depression and anxiety (Rollins & Crandall, 2021). In a study using Australian data, Kemp et al. (2024) found that for each increase in the positive youth experience score, the odds of having good mental health—defined as a score of 18 or less on the 6‐item Kessler Psychological Distress Scale (K6)—increased by 28% (OR = 1.28, 95% CI: 1.14–1.44, *p* < 0.001), reflecting lower levels of overall psychological distress (Kemp et al., 2024). Gondek et al. (2023) found strong evidence of an association between cumulative positive experiences and depressive symptoms at age 18, with each additional highly positive experience across 11 domains associated with a 4.1% reduction in Short Mood and Feelings Questionnaire ,SMFQ scores (*β* = −0.042, 95% CI −0.060 to −0.025) (Gondek et al., 2023). At the domain level, nearly all (10/11) positive experiences were associated with lower depressive symptoms in adjusted models, although individual effect sizes were small (0.1%–2.7% reduction in SMFQ scores per unit increase) (Gondek et al., 2023). Overall, evidence suggests that greater exposure to cumulative positive experiences is associated with lower levels of depression and anxiety, as well as better overall mental health, although effect sizes are generally modest.

## DISCUSSION

To our knowledge, this is the first systematic review of longitudinal cohort studies examining the association between a broad range of positive experiences, including both singular and cumulative exposures, and adult mental health outcomes specifically. Findings across the 20 included studies were mixed, with several demonstrating statistically significant associations between positive experiences and reduced risk of depression, anxiety, or psychotic symptoms, while others reported null effects, likely reflecting heterogeneity in measurement, timing, and outcomes. Notably, 12 studies (Allen et al., 2024; Cong et al., 2020; Crandall et al., 2020; Crush et al., 2018; Glickman et al., 2021; Gondek et al., 2023; Kandola et al., 2020; Kemp et al., 2024; Nyberg et al., 2019; Sormunen et al., 2017; Steiner et al., 2019; Upenieks & Thomas, 2021) retained statistical significance after adjustment for confounders, and eight of these were rated as high quality.

The most frequently examined exposure was physical activity, with several studies showing associations with lower depression scores in adulthood (da Silva et al., 2023; Howie et al., 2020; Kandola et al., 2020). Measurement of physical activity varied across studies, with one study using an objective measure (i.e., accelerometery) while others primarily relied on self‐report. Regardless of measurement approach, physical activity in childhood and adolescence typically occurs within school or other group‐based contexts. Within this review, it was therefore conceptualised as an indicator of participation in structured or socially embedded developmental contexts, rather than solely as an isolated health behaviour. Despite heterogeneity in measurement, findings for depression were broadly consistent, supporting the robustness of the association. Notably, the two studies that did not retain statistical significance (Brunet et al., 2013; McKercher et al., 2014) both relied on self‐reported physical activity; given the known imprecision and potential for non‐differential misclassification in self‐report measures, this attenuation following adjustment may partly reflect measurement‐related bias, although residual confounding and population differences cannot be ruled out.

Other positive experiences, including engaged parenting (Cong et al., 2020), perceived social support from peers and family (Glickman et al., 2021; Låftman et al., 2025), school belonging (Allen et al., 2024), school and family connectedness (Steiner et al., 2019), and religious service attendance (Upenieks & Thomas, 2021), were also linked to improved mental health outcomes. A positive home atmosphere and higher levels of social cohesion were associated with reduced risk of psychotic symptoms (Crush et al., 2018), while school connectedness (Nyberg et al., 2019) and social support (Låftman et al., 2025) were linked to lower adult anxiety. These findings align with a substantial body of meta‐analytic evidence demonstrating that parenting dimensions such as sensitivity, responsiveness, and warmth are robustly associated with the development of secure attachment (De Wolff & van Ijzendoorn, 1997; Lucassen et al., 2011; Madigan et al., 2024; Santana‐Ferrándiz et al., 2025). In turn, attachment security has been consistently linked to more positive developmental outcomes and lower risk of a broad range of socioemotional, behavioural, and cognitive difficulties across the life course (Cooke et al., 2022; Costa Martins et al., 2022; Groh et al., 2014, 2017; Rodrigues et al., 2021), underscoring the central role of early caregiving environments in shaping long‐term mental health trajectories. Taken together, these findings suggest that positive experiences characterised by social connection and support—across family, peer, school, and community contexts—may be particularly relevant for later mental health, rather than any single exposure in isolation.

Overall, these findings align with previous reviews that have highlighted the promotive and protective potential of positive experiences. For example, Han et al. (2023) found that higher levels of positive experiences were associated with improved mental health and psychosocial outcomes (Han et al., 2023). Similarly, Cunha et al. (2024) reported associations between positive experiences and reduced symptoms of depression, anxiety, post‐traumatic stress disorder, and other mental health indicators (Cunha et al., 2024). However, most prior reviews have focussed on adversity‐exposed samples or retrospective measures from cross‐sectional studies, which limits generalisability and causal inference.

As is often the case with ACEs, positive experiences seldom occur in isolation, leading researchers to emphasise the importance of accounting for their accumulation across multiple ecological levels (Han et al., 2023). Although we included studies examining singular exposures to provide insight into which relational or environmental contexts are most consistently associated with adult mental health outcomes, such approaches may not fully capture the cumulative and multilevel nature of positive experiences and may consequently influence effect size estimates, as isolating interrelated exposures can obscure their shared and cumulative contributions to mental health outcomes. While we were only able to identify four eligible longitudinal studies that used a cumulative measure of positive experiences (Crandall et al., 2020; Gondek et al., 2023; Kemp et al., 2024; Rollins & Crandall, 2021), results showed broadly consistent associations with better mental health outcomes despite relatively modest effect sizes. These findings suggest that both singular and cumulative positive experiences exert broadly promotive effects on mental health, with cumulative exposure providing benefit irrespective of adversity exposure.

### Strengths and limitations

A key strength of the included studies is their prospective design with long‐term follow‐up, enhancing confidence in the temporal ordering of childhood exposures and adult mental health outcomes. However, this review should be interpreted in light of some limitations. First, although we employed a systematic search strategy across multiple databases, it is possible that relevant longitudinal studies examining conceptually similar singular positive experiences were missed due to variability in terminology and our operationalisation of positive experiences. This limitation highlights conceptual fragmentation within the field and underscores the need for greater standardisation of definitions and measures for both discrete and cumulative positive experiences (Han et al., 2023; Raghunathan et al., 2024). Relatedly, attachment security and general parenting constructs (e.g., parenting style typologies) were not considered eligible exposures unless explicitly framed as positive experiential assets. Consequently, some longitudinal research examining these constructs in relation to later mental health outcomes may not be represented. This reflects a deliberate conceptual boundary of the review rather than a true absence of evidence.

Second, the heterogeneity of included studies prevented meta‐analysis and limited our ability to conduct subgroup analyses. Variations in exposure measurement, outcome definition, and analytic approaches may have influenced the comparability of findings (Bogdan et al., 2023). Third, many studies relied on self‐ or parent‐report, which may be subject to social desirability and recall bias. More objective or multi‐informant approaches to assessing positive experiences, particularly within family and school domains, would strengthen future research. Fourth, while all included studies were longitudinal, most relied on observational designs, which restrict causal inference. Residual confounding and measurement bias remain possible, particularly in studies using self‐reported exposures.

Fifth, this review focussed on select adverse mental health outcomes and did not consider other important indicators such as physical health, psychosocial and positive mental health outcomes (e.g., flourishing). This limits the generalisability of findings to broader aspects of well‐being. Sixth, nearly all studies were conducted in high‐income countries, which constrains the applicability of results. Research in low‐ and middle‐income contexts is needed to understand how positive experiences operate across diverse cultural and socioeconomic contexts (Cunha et al., 2024; Kallapiran et al., 2025). Finally, this review did not examine interactions between positive experiences and adversity. Consequently, we cannot determine whether observed associations reflect protective effects.

### Implications and future research

The findings of this review highlight the potential value of positive experiences in childhood and adolescence for promoting mental health across the life course. While evidence remains mixed, several modifiable experiences, including physical and leisure‐time activities, engaged parenting, supportive peer and family relationships, and aspects of school belonging and connectedness, emerged as promising targets for early intervention. Public health strategies and educational policies that foster these experiences may help reduce the prevalence and severity of mental health problems in adulthood.

Future research should address key gaps identified in this review. First, there is a need for greater conceptual clarity and consistency in defining and prospectively measuring positive experiences. Broader adoption of validated instruments, such as the BCEs scale (Narayan et al., 2018) and the modified HOPE framework (Guo et al., 2022), within longitudinal cohorts would enable more robust comparisons across studies and help clarify the cumulative impact of positive experiences on long‐term mental health outcomes. Second, longitudinal research should examine the timing and duration of exposure to positive experiences to determine whether certain developmental periods confer greater benefits. Third, research should extend beyond high‐income countries to include culturally and socioeconomically diverse populations, ensuring that interventions are equitable and globally relevant (Cunha et al., 2024).

Finally, future work should broaden the scope of outcomes to include indicators of positive mental health, such as flourishing and life satisfaction, alongside the prevention of mental illness. Understanding how positive experiences contribute to overall well‐being will provide a more comprehensive foundation for designing interventions that support healthy development throughout the lifespan.

## CONCLUSION

This review provides evidence that positive experiences in childhood, such as physical activity, supportive parenting, peer relationships, social support, and school belonging, may be associated with improved mental health outcomes in adulthood. While findings were mixed, several experiences demonstrated relatively consistent associations with reduced risk of depression, anxiety, and psychotic symptoms. However, the interpretation of this evidence is shaped by considerable definitional and terminological heterogeneity within the field, as well as variation in how exposures are operationalised across studies. As such, the apparent scope and distribution of the evidence base may reflect these conceptual differences as much as underlying gaps in the literature.

Notwithstanding these considerations, investing in strategies that foster positive experiences during childhood and adolescence across family, school, and community contexts may still offer a promising avenue for reducing the burden of mental illness. Such efforts have the potential to improve population mental health and support healthier developmental trajectories. Continued research is needed to strengthen the evidence base and to improve conceptual clarity and consistency in how positive experiences, particularly cumulative indices, are defined and measured in longitudinal studies.

## AUTHOR CONTRIBUTIONS

**Lisa H. Telford**: Methodology (lead); investigation (lead); data curation (lead); formal analysis (lead); visualization (lead); project administration (equal); writing—original draft (lead); writing—review & editing (equal). **Colm Healy**: Supervision (supporting); writing—review & editing (equal). **Éilis J. O'Reilly**: Supervision (supporting); writing—review & editing (equal). **Lama Alquthami**: Data curation (supporting); Investigation (supporting). **Amna Al Hitmi**: Investigation (supporting); data curation (supporting). **Sami Almasri**: Data curation (supporting); investigation (supporting). **Mary C. Clarke**: Conceptualization (lead); investigation (supporting); data curation (supporting); project administration (equal); supervision (lead); writing—review & editing (equal).

## CONFLICT OF INTEREST STATEMENT

The authors declare no conflicts of interest.

## ETHICAL CONSIDERATIONS

Ethical approval is not applicable to this article as no new data were collected during the review process.

## Supporting information

Supporting Information S1

## Data Availability

The data that support the findings of this study are available from the corresponding author upon reasonable request.
