# Supplementary material for: The impact of positive experiences in childhood and adolescence on later mental health: A systematic review of longitudinal cohort studies
Source: JCPP Adv. 2026 Nov 13:e70172. Online ahead of print. doi: 10.1002/jcv2.70172 (PMC13765551; doi:10.1002/jcv2.70172)
Supplement: Supplementary file 1 [file JCV2-9999-e70172-s001.docx]

**The impact of positive experiences in childhood and adolescence on later mental health: A systematic review of longitudinal cohort studies**

**Supporting Information**

**Table S1.** *Inclusion and exclusion criteria*

| **Category** | **Inclusion** | **Exclusion** |
| --- | --- | --- |
| Language | - Written in the English language. | - Not written in the English language, or only the title and abstract are written in English. |
| Publication Type | - Peer-reviewed studies/articles. | - Sources which do not report on or include data analysis, such as review articles, book chapters or conference papers. |
| Study Characteristics / Design | - Prospective or retrospective cohort studies investigating associations between exposure to positive experiences and the development of mental illness (i.e., anxiety, depression, bipolar and psychotic disorder/symptoms or psychopathology) in adulthood. | - Review articles, randomised control trials, editorials, conference abstracts, cross-sectional studies. |
| Study Population | - Humans. - All participants from the general population aged 18 or younger at the time of exposure. | - Animal studies/models. - Studies using selected or clinical high-risk samples. |
| Intervention / Exposure | - Exposure to singular (one safe caregiver, friends, comforting beliefs, school enjoyment, a caring teacher, good neighbours, an adult mentor, opportunities, positive self-image, home routines, community traditions, spiritual beliefs, education, school belonging, skills development, household safety, physical activity, and hobbies) or cumulative positive experiences within the first 18 years of life (≤ 18 years) and measured prior to the development of the specified outcome(s). | - Studies where exposure was assessed after age 18 (i.e., in adulthood). |
| Comparator / Context | - Studies reporting risk for mental health outcomes among those who have had positive experience exposure compared to risk in the unexposed population group. |  |
| Outcome | - A clinical diagnosis of anxiety disorder, mood disorder (including major depressive disorder or bipolar disorder), or psychotic disorder based on Diagnostic and Statistical Manual of Mental Disorders (DSM) or International Classification of Diseases (ICD) criteria, or measurement of symptoms of these disorders or broader psychopathology using a validated instrument in adulthood (>18 years). | - Studies where outcomes were measured before the age of 18 (in childhood). - Studies recording ‘lifetime’ diagnosis in adulthood. |

**Table S2.** *Search strategy and results*

| **APA PsycInfo** | **Original Search** | **Updated Search** |
| --- | --- | --- |
| ( ( DE "Mental Disorders" OR "mental disorders" OR “mental disorder” OR “mental symptoms” OR “mental health symptoms” OR “mental health outcomes” OR “psychiatric disorder” OR “psychiatric symptoms” OR “psychopathology” OR “psychopathology symptoms” OR DE "Psychopathology" ) OR ( DE "Anxiety Disorders" OR “anxiety disorders” OR “anxiety disorder” OR "anxiety disorder symptoms" ) OR ( DE "Affective Disorders" OR “mood disorder” OR “mood disorders”  OR "mood disorder symptoms" ) OR ( DE "Major Depression" OR DE "Disruptive Mood Dysregulation Disorder" OR (major N2 depression) OR (major N2 depressive) OR  "major depression symptoms" ) OR ( DE "Bipolar Disorder" OR “bipolar disorder" OR (bipolar N3 disorder$?) OR (bipolar N1 depression) OR (bipolar N1 mania) or cyclothymia or "bipolar disorder symptoms" ) OR ( DE "Psychosis" OR DE "Acute Psychosis" OR DE "Affective Psychosis" OR DE "Chronic Psychosis" OR TI psychosis OR AB psychosis  OR "psychotic symptoms" ) ) AND ( ( TI ( ("positive childhood experiences" or "advantageous childhood experiences" or "benevolent childhood experiences" or "protective and compensatory experiences" or "protective childhood experiences" or "promotive childhood experiences" or "protective factors" or "promotive factors" or "counter-ACEs" or resilience or "resilience factors") ) OR AB ( ("positive childhood experiences" or "advantageous childhood experiences" or "benevolent childhood experiences" or "protective and compensatory experiences" or "protective childhood experiences" or "promotive childhood experiences" or "protective factors" or "promotive factors" or "counter-ACEs" or resilience or "resilience factors") ) OR DE "Protective Factors" OR DE "Resilience (Psychological)" ) OR ( TI ("Caregiver" or "caregivers" or "caregiving" or "friends" or "best friend" or "comforting beliefs" or "school enjoyment" or "caring teacher" or "teacher support" or "neighbor$" or "mentor" or "opportunities" or "positive self-image" or "home routine$" or "community traditions" or "spiritual beliefs" or "education" or "school belonging" or "skills development" or "household safety" or "physical activity" or "hobbies") ) ) AND ( DE "Longitudinal Studies" OR "longitudinal studies" OR "longitudinal study" OR DE "Prospective Studies" OR "Prospective Studies" )  *Limited to English  *Excluding animal studies, systematic reviews and meta-analyses | 1042 records | 1419 records |
| **Embase** | **Original Search** | **Updated Search** |
| ('mental disorder'/de OR 'mental disorder':ti,ab,kw OR 'mental symptoms':ti,ab,kw OR 'mental health symptoms':ti,ab,kw OR 'mental health outcomes':ti,ab,kw OR 'psychiatric disorder':ti,ab,kw OR 'psychiatric symptoms':ti,ab,kw OR 'psychopathology':ti,ab,kw OR 'psychopathology symptoms':ti,ab,kw OR 'anxiety disorder'/de OR 'anxiety disorder':ti,ab,kw OR ((anxiety NEXT/1 disorder):ti,ab,kw) OR ((anxiety NEXT/1 disorders):ti,ab,kw) OR 'anxiety disorder symptoms':ti,ab,kw OR 'mood disorder'/de OR ((mood NEXT/1 disorder):ti,ab,kw) OR ((mood NEXT/1 disorders):ti,ab,kw) OR 'mood disorder symptoms':ti,ab,kw OR 'major depression'/de OR ((major NEXT/2 depression):ti,ab,kw) OR ((major NEXT/2 depressive):ti,ab,kw) OR 'major depression symptoms':ti,ab,kw OR 'bipolar disorder'/de OR ((bipolar NEXT/3 disorder$):ti,ab,kw) OR ((bipolar NEXT/1 depression):ti,ab,kw) OR ((bipolar NEXT/1 mania):ti,ab,kw) OR cyclothymia:ti,ab,kw OR 'bipolar disorder symptoms':ti,ab,kw OR 'psychosis'/de OR 'psychosis':ti,ab,kw OR 'psychotic':ti,ab,kw OR 'psychotic symptoms':ti,ab,kw) AND ('positive childhood experiences':ti,ab OR 'advantageous childhood experiences':ti,ab OR 'benevolent childhood experiences':ti,ab OR 'protective and compensatory experiences':ti,ab OR 'protective childhood experiences':ti,ab OR 'promotive childhood experiences':ti,ab OR 'protective factors':ti,ab OR 'promotive factors':ti,ab OR 'counter-aces':ti,ab OR 'resilience':ti,ab OR 'resilience factors':ti,ab OR 'caregiver':ti OR 'caregivers':ti OR 'caregiving':ti OR 'friends':ti OR 'best friend':ti OR 'comforting beliefs':ti OR 'school enjoyment':ti OR 'caring teacher':ti OR 'teacher support':ti OR 'neighbor$':ti OR 'mentor':ti OR 'opportunities':ti OR 'positive self-image':ti OR 'home routines':ti OR 'community traditions':ti OR 'spiritual beliefs':ti OR 'education':ti OR 'school belonging':ti OR 'skills development':ti OR 'household safety':ti OR 'physical activity':ti OR 'hobbies':ti) AND ('longitudinal study'/de OR 'longitudinal study':ti,ab OR 'longitudinal studies':ti,ab OR 'cohort analysis'/de OR 'cohort analysis':ti,ab OR 'cohort study':ti,ab OR 'cohort studies':ti,ab OR 'prospective study'/de OR 'prospective study':ti,ab OR 'prospective studies':ti,ab) NOT [medline]/lim AND [english]/lim AND ('article'/it OR 'article in press'/it OR 'conference abstract'/it OR 'conference review'/it OR 'erratum'/it OR 'note'/it OR 'preprint'/it) | 667 records | 1050 records |
| **Medline** | **Original Search** | **Updated Search** |
| (Mental Disorder/ or ("mental disorders" or "mental disorder").mp. or "mental symptoms".mp. or "mental health symptoms".mp. or "mental health outcomes".mp. or "psychiatric disorder".mp. or "psychiatric symptoms".mp. or "psychopathology".mp. or "psychopathology symptoms".mp. or Psychopathology/ or Anxiety Disorders/ or ("anxiety disorders" or "anxiety disorder").mp. or "anxiety disorder symptoms".mp. or Mood Disorders/ or ("mood disorder" or "mood disorders").mp. or "mood disorder symptoms".mp. or Depressive Disorder, Major/ or (major adj2 depression).mp. or (major adj2 depressive).mp. or "major depression symptoms".mp. or Psychotic Disorders/ or psychosis.mp. or psychotic.mp. or "psychotic symptoms".mp.) and (("positive childhood experiences" or "advantageous childhood experiences" or "benevolent childhood experiences" or "protective and compensatory experiences" or "protective childhood experiences" or "promotive childhood experiences" or "protective factors" or "promotive factors" or "counter-ACEs" or resilience or "resilience factors").tw. or Protective Factors/ or Resilience, Psychological/ or ("caregiver" or "caregivers" or "caregiving" or "friends" or "best friend" or "comforting beliefs" or "school enjoyment" or "caring teacher" or "teacher support" or "neighbor$" or "mentor" or "opportunities" or "positive self-image" or "home routine$" or "community traditions" or "spiritual beliefs" or "education" or "school belonging" or "skills development" or "household safety" or "physical activity" or "hobbies").ti.) and (Longitudinal Studies/ or longitudinal.mp. or ("cohort study" or "cohort studies").mp. or Cohort Studies/ or "cohort analysis".mp. or Prospective Studies/ or prospective studies.mp.)  *Limit to English language  *Limit to humans only (removes records about animals) | 1623 records | 1780 records |
| **Scopus** | **Original Search** | **Updated Search** |
| ((TITLE-ABS-KEY ( "mental disorder" OR "mental disorders" OR "mental symptoms" OR "mental health symptoms" OR "mental health outcomes" OR "psychiatric disorder" OR "psychiatric symptoms" OR "psychopathology" OR "psychopathology symptoms" )) OR (TITLE-ABS-KEY ( "anxiety disorder" OR "anxiety disorders" OR "anxiety disorder symptoms" )) OR (TITLE-ABS-KEY ( "mood disorder" OR "mood disorders" OR "mood disorder symptoms" )) OR (TITLE-ABS-KEY ( ( major W/2 depression ) OR ( major W/2 depressive ) OR "major depression symptoms" )) OR (TITLE-ABS-KEY ( "bipolar disorder" OR ( bipolar W/3 disorder$ ) OR ( bipolar W/1 depression ) OR ( bipolar W/1 mania ) OR cyclothymia OR "bipolar disorder symptoms" )) OR (TITLE-ABS-KEY ( psychosis OR psychotic OR "psychotic symptoms" ))) AND ((TITLE-ABS-KEY ( "positive childhood experiences" OR "advantageous childhood experiences" OR "benevolent childhood experiences" OR "protective and compensatory experiences" OR "protective childhood experiences" OR "promotive childhood experiences" OR "protective factors" OR "promotive factors" OR "counter-aces" OR "resilience" OR "resilience factors" )) OR (TITLE ( "Caregiver" OR "caregivers" OR "caregiving" OR "friends" OR "best friend" OR "comforting beliefs" OR "school enjoyment" OR "caring teacher" OR "teacher support" OR "neighbor$" OR "mentor" OR "opportunities" OR "positive self-image" OR "home routine$" OR "community traditions" OR "spiritual beliefs" OR "education" OR "school belonging" OR "skills development" OR "household safety" OR "physical activity" OR "hobbies" ))) AND (TITLE-ABS ( "longitudinal study" OR "longitudinal studies" OR "cohort analysis" OR "cohort study" OR "cohort studies" OR "prospective study" OR "prospective studies" )) AND ( LIMIT-TO ( LANGUAGE,"English" ) ) AND ( EXCLUDE ( DOCTYPE,"re" ) ) | 1065 records | 1490 records |

**Table S3.** *Data extraction items*

| **Category** | **Items** |
| --- | --- |
| Study Details | Authors |
|  | Report title |
|  | Journal name |
|  | Publication year |
|  | Study design |
|  | Dataset (cohort) used |
|  | Study start and end date |
|  | Duration of study period |
|  |  |
| Population and Setting | Country of study |
|  | Participant inclusion and exclusion criteria |
|  | Method of recruitment |
|  | Analytic sample size |
|  | Proportion or number of participants by sex |
|  | Race/ethnicity |
|  |  |
| Exposure Assessment | Definition of exposure(s) |
|  | Exposure measurement method |
|  | Age(s) at which exposure(s) measured |
|  | Exposure categories or levels |
|  | Frequency or duration of exposure |
|  | Confounding factors considered or adjusted for |
|  |  |
| Outcome Assessment | Definition of outcome(s) |
|  | Outcome measurement method |
|  | Age(s) at which outcome(s) measured |
|  | Loss to follow-up or attrition rate |
|  |  |
| Results | Statistical analysis type |
|  | Effect estimate type |
|  | Effect estimate and corresponding 95% confidence interval (CI) or Standard Error (SE) |
|  | Statistical significance (p-value) |
|  | Direction of association |
|  | Interpretation of result |
|  | Subgroup analyses or effect modification |
|  | Key conclusions of study authors |
|  | Strengths and limitations of the study |
|  | Recommendations |
|  |  |
| Other Information | References to other potentially relevant studies |
|  | Study funding sources |
|  | Conflicts of interest |
|  |  |

**Table S4.** *List of studies excluded after full-text review*

This table includes studies excluded at full-text screening from both the original search conducted on 26 January 2024 and the updated search conducted on 20 May 2026.

| **Study citation** | **Search phase** | **Reason for exclusion** |
| --- | --- | --- |
| **Identified via databases and registers (original search n = 21; updated search n = 37)** | | |
| Bang et al., 2024 | Updated search | **Outcome measured in childhood:** Depression measured in adolescence (before age 18). |
| Baran et al., 2025 | Updated search | **Exposure measured in adulthood:** Personality, spiritual fitness, and positive mental health assessed in adulthood. |
| Bates et al., 2023 | Updated search | **Wrong outcome:** Prosocial behaviour falls outside the scope of eligible outcomes nor was it measured in adulthood. |
| Bayar, 2025 | Updated search | **Wrong study design:** Compares the BCE and BCE-Revised scales in terms of their psychometric properties. |
| Brieant and Burt, 2025 | Updated search | **Outcome measured in childhood:** Outcomes measured at year three follow up when participants would have been aged 12-13 years. |
| Bromley et al., 2024 | Updated search | **Outcome measured in childhood:** Psychological distress and wellbeing measured in childhood (before age 18). |
| Brown et al., 2025 | Updated search | **Wrong study design:** Uses data from online survey responses and focus groups and is therefore mixed-methods and not longitudinal. |
| Buchanan et al., 2024 | Updated search | **Exposure measured in adulthood:** Social support assessed in adulthood, at ages 18, 21, 25, 30, 35 and 40, rather than in childhood. |
| Cahill et al., 2022 | Original search | **Wrong exposure:** Resilience measured using the residuals approach and not through primary measurement of resilience exposure using a scale. |
| Cammack and Suglia, 2023 | Original search | **Selected/high-risk sample:** The study sample was selected based on exposure to any child maltreatment making them a high-risk group and not representative of the general population. |
| Cárdenas et al., 2022 | Original search | **Selected/high-risk sample:** The study sample (pregnant women) are a selected sample and not part of the general population. |
| Chen et al., 2020 | Original search | **Wrong exposure:** No relevant positive experiences measured before the age of 18. |
| Corallo et al., 2023 | Original search | **Outcome not measured using a standardised instrument:** Mental health not measured using a standardised instrument. |
| Dibben et al., 2017 | Original search | **Exposure measured in adulthood:** Exposure (participation in Scouts-Guides) measured at the same time as the outcome in adulthood (age 50). |
| Easterlin et al., 2019 | Original search | **Selected/high-risk sample:** The analytic sample was selected based on having had any adversity exposure and is therefore high-risk and not representative of the general population. |
| Fitzpatrick et al., 2024 | Updated search | **Outcome measured in childhood:** Uses wave 1 survey data when participants were aged 11-17 with a mean age of 14 years. |
| Galera et al., 2025 | Updated search | **Wrong exposure:** Neighbourhood green space is not considered an eligible exposure. |
| Ganjeh et al., 2024 | Updated search | **Outcome measured in childhood:** Psychopathology measured using the SDQ assessed in childhood (before age 18). |
| Garnsey et al., 2026 | Updated search | **Exposure measured in adulthood:** Trait self-compassion measured in adulthood - sample population mean age of 19 years at baseline. |
| Godoy-Casasbuenas et al., 2025 | Updated search | **Selected/high-risk sample:** Uses a sample of adolescents and young adults from the most disadvantaged neighbourhoods. |
| Hardi et al., 2026 | Updated search | **Outcome measured in childhood:** Mental health symptoms measured at various timepoints all in childhood (before age 18). |
| Herath and Zhang, 2025 | Updated search | **Exposure measured in adulthood:** Parent-child relationships measured in adulthood at Wave IV when participants were aged 24-32. |
| Huynh et al., 2025 | Updated search | **Wrong study design:** Uses a cross-sectional design. |
| Iyer et al., 2023 | Original search | **Lifetime diagnosis and selected/high-risk sample:** Uses a composite measure of mental health outcomes which includes both current depressive symptoms and/or lifetime mental health diagnoses. The study also used a selected sample of Asian Americans from a larger national longitudinal cohort. |
| Kim et al., 2024 | Updated search | **Exposure measured in adulthood:** Perceived neighbourhood social cohesion measured when participants ranged in age from 12 to 21 years therefore some had exposure in adulthood. |
| Kingsbury et al., 2020 | Updated search | **Outcome measured in childhood:** Mental health symptoms measured in childhood at T1 (aged 12/13) and T2 (ages 14/15). |
| König et al., 2025 | Updated search | **Selected/high-risk sample:** Uses a sample of young people with childhood adversity. |
| Latham et al., 2022 | Original search | **Selected/high-risk sample:** Relevant investigations using eligible exposures and outcomes were conducted in a violence exposed (at-risk) subgroup. |
| Lewis and McKelvey, 2025 | Updated search | **Outcome measured in childhood:** Outcomes measured when participants were in Grade 5 (age 11) therefore in middle childhood. |
| Lim, 2025 | Updated search | **Exposure measured in adulthood:** Physical activity measured in adulthood rather than during childhood/adolescence. |
| Lundgren et al., 2025 | Updated search | **Outcome measured in childhood:** Psychiatric diagnoses were collected from birth to age 18. |
| Maurer et al., 2025 | Updated search | **Wrong exposure:** Primary exposure is socioeconomic status rather than a positive childhood experience. |
| McPhie and Rawana, 2015 | Original search | **Outcome measured from childhood:** Mental health outcome modelled as a developmental trajectory spanning adolescence into adulthood (ages 15–28). |
| Miller et al., 2024 | Updated search | **Wrong study design:** Is a systematic review. |
| Mondi et al., 2020 | Original search | **Selected/high-risk sample:** Selected sample from low-income communities therefore not representative of the general population. |
| O’Reilly et al., 2022 | Original search | **Wrong outcome:** Adolescent substance use and self-harm behaviour (assessed at age 18) not within eligibility criteria. |
| Patten et al., 2009 | Original search | **Outcome measured in childhood:** Outcome of interest, major depressive episode, measured at every timepoint beginning at baseline when participants were 12 or older (and every 2 years thereafter) therefore measured in childhood (before age 18). |
| Pérez Rodríguez et al., 2024 | Updated search | **Wrong outcome:** Suicidal ideation falls outside the scope of eligibility criteria. |
| Ranøyen et al., 2025 | Updated search | **Selected/high-risk sample:** Uses a clinical sample of adolescents with depression or anxiety disorders. |
| Ren et al., 2024 | Updated search | **Exposure measured in adulthood:** Family environment measured in a sample of postgraduate students therefore in adulthood. |
| Rueger and George, 2017 | Original search | **Outcome measured in childhood:** Depressive symptoms measured in adolescence, before participants were 18 years old, criteria required mental health outcomes in adulthood (age >18). |
| Sahib et al., 2025 | Updated search | **Outcome measured in childhood:** Adolescent mental health assessed at Wave 3 when participants were in Grades 9 and 10. |
| Salvatore et al., 2011 | Original search | **Dissertation:** Unpublished (grey) literature. |
| Shoshani, 2024 | Updated search | **Outcome measured in childhood:** Mental health and well-being in childhood/adolescence and not in adulthood. |
| Skodol et al., 2007 | Original search | **Wrong outcome:** Outcomes examined (schizotypal, borderline, avoidant, or obsessive-compulsive personality disorders) fall outside the scope of eligibility criteria. |
| Stavrakakis et al., 2013 | Original search | **Outcome measured in childhood:** Major depressive episode (MDE) includes any episode between T2 (age 13) and T4 (age 18), criteria required outcomes in adulthood (age >18) only. |
| Stock et al., 2023 | Original search | **Abstract:** Abstract only. |
| Suetani et al., 2017 | Original search | **Lifetime diagnosis:** Lifetime diagnosis of common mental disorders measured using the Youth Self Report (YSR) at age 14 and at age 21. |
| Suetani et al., 2018 | Original search | **Abstract:** Abstract only. |
| Svensson et al., 2021 | Updated search | **Exposure measured in adulthood:** Physical activity measured in adulthood – sample had a median age of 36 years at baseline. |
| Tietbohl-Santos et al., 2025 | Updated search | **Selected/high-risk sample:** Uses a high-risk sample of young adults, including individuals with a familial predisposition, maltreatment history, and socioeconomic vulnerabilities. |
| Tort-Carrera et al., 2026 | Updated search | **Exposure measured in adulthood:** Neighbours and neighbourhood exposure assessed in adulthood and not in childhood. |
| Tutzer et al., 2024 | Updated search | **Selected/high-risk sample:** Uses a sample of participants with pre-existing mental health disorders. |
| VanBronkhorst et al., 2024 | Original search | **Outcome measured in childhood:** Outcome (perceived stress and mental disorders) assessed at wave 4 when participants ranged in age from 15 to 29 years, criteria required outcomes in adulthood (age >18) only. |
| Wang and Wang, 2026 | Updated search | **Outcome measured in childhood:** Mental health (emotional and peer problems) assessed in childhood and adolescence. |
| Wang et al., 2025 | Updated search | **Exposure measured in adulthood:** Physical activity measured in adulthood (after age 18) and not in childhood. |
| Wolke et al., 2025 | Updated search | **Wrong study design:** Is a systematic review. |
| Wong et al., 2026 | Updated search | **Wrong exposure:** Loneliness, social isolation, and social connection fall outside the scope of eligibility criteria. |
| **Identified via other methods (original search n = 31; updated search n = 28)** | | |
| Agerup et al., 2015 | Original search | **Outcome measured in childhood:** Depression measured at age 15, criteria required adult outcomes (age >18). |
| Ahrens et al., 2008 | Original search | **Selected/high-risk sample:** Sample were youth who were ever in foster care therefore high-risk and not representative of the general population. |
| Banyard et al., 2008 | Updated search | **Selected/high-risk sample:** Uses a selected convenience sample of women obtaining social services for family violence. |
| Bethell et al., 2019 | Original search | **Wrong study design:** The study used a cross-sectional survey design rather than a longitudinal cohort. |
| Chen and Harris, 2019 | Updated search | **Outcome measured in childhood:** Study models trajectories of depressive symptoms starting in adolescence. |
| Chen and VanderWeele, 2018 | Updated search | **Selected sample and exposure measured into adulthood:** Study uses data from the Nurses’ Health Study II (NHSII), a cohort of nurses, and the Growing Up Today Study (GUTS), children of NHSII participants. Religious involvement assessed when GUTS participants were aged 12-19 years. |
| Chung et al., 2008 | Original search | **Selected/high-risk sample:** The study used a sample of low-income pregnant women which is not representative of the general population. |
| Clements-Nolle and Waddington, 2019 | Original search | **Wrong study design and selected/high-risk sample:** The study used a cross-sectional design and the sample were juvenile offenders. |
| Crandall et al., 2019 | Original search | **Wrong study design:** The study used data from an online survey (cross-sectional) rather than a longitudinal cohort design. |
| Delawalla, 2019 | Updated search | **Dissertation:** Unpublished (grey) literature. |
| Eshel et al., 2006 | Original search | **Wrong study design:** The study was a systematic review of the literature rather than a longitudinal cohort study. |
| Fang et al., 2024 | Updated search | **Exposure measured in adulthood:** Parental warmth not restricted to childhood, includes concurrent adult exposure. |
| Fitzgerald and Gallus, 2020 | Updated search | **Exposure measured in adulthood:** Emotional support from family, friends, and romantic partners measured in adulthood. |
| Goodrum et al., 2020 | Updated search | **Wrong exposure:** Family cohesion treated as a broad family functioning variable within a risk–behaviour framework rather than a discrete positive experience. |
| Gunnarsdóttir et al., 2021 | Updated search | **Wrong exposure:** Poor school connectedness falls outside the scope of eligible positive experiences. |
| Hays-Grudo et al., 2021 | Original search | **Wrong study design:** This paper proposes a model based on existing knowledge rather than an empirical longitudinal cohort study. |
| Herman et al., 2024 | Updated search | **Wrong outcome:** Young adult well-being falls outside the scope of eligible outcomes. |
| Herrenkohl et al., 2016 | Updated search | **Selected/high-risk sample:** Study sample are a child maltreatment–exposed cohort. |
| Huang et al., 2023 | Updated search | **Exposure measured in adulthood:** Positive experiences measured via retrospective recall in adulthood. |
| Hurd and Zimmerman, 2010 | Original search | **Selected/high-risk sample:** The study sample (African Americans) was selected from a larger more representative cohort. |
| Hurd and Zimmerman, 2014 | Updated search | **Wrong exposure:** Mentoring relationship profiles/social support constructs rather than discrete positive experiences. |
| Jacka et al., 2011 | Original search | **Lifetime diagnosis:** The study outcome was lifetime history of self-reported depression, criteria required outcomes measured in adulthood. |
| Jerstad et al., 2010 | Original search | **Selected/high-risk sample:** The study sample were adolescent girls only, and considered a high-risk population. |
| Kendler et al., 2005 | Original search | **Exposure measured in adulthood:** Exposure (social support) measured at wave 1 which was on average 19 months before wave 2 when participants were between 21 and 58 years old, therefore social support was measured in adulthood, criteria required exposure in childhood (age ≤ 18). |
| Kent and Bradshaw, 2021 | Updated search | **Outcome measured in childhood:** Outcomes not restricted to adulthood; depressive symptom trajectories span adolescence into adulthood. |
| Kim et al., 2022 | Updated search | **Exposure measured in adulthood:** Self-esteem assessed 7 years after baseline (when participants were in grades 7-12). |
| Knappe et al., 2009a | Original search | **Wrong outcome:** The outcome examined (social phobia) was not within the scope of eligible outcomes. |
| Knappe et al., 2009b | Original search | **Wrong exposure:** The exposures examined (parental psychopathology, parental rearing, and family functioning) were risk factors rather than positive experiences which are protective and/or promotive factors. |
| Knappe et al., 2009c | Original search | **Wrong outcome and exposures:** The outcome examined (social phobia) was not within the scope of eligible outcomes and exposures (parental rearing and family functioning) were conceptualised as risk factors. |
| Korhonen et al., 2009 | Original search | **Wrong outcome:** The outcomes examined (alcohol and illicit drug use) were not within the scope of eligible mental health outcomes. |
| Kosterman et al., 2011 | Original search | **Wrong outcome:** The outcomes examined (positive adult functioning, including civic involvement, productivity and responsibility, interpersonal connection, and physical exercise) fell outside the scope of eligible outcomes. |
| Luby et al., 2019 | Original search | **Wrong outcome:** The outcome (structural brain development) was not within the scope of eligible outcomes. |
| Lyu and Agrigoroaei, 2017 | Updated search | **Exposure measured in adulthood:** Quality of social relations measured at Time 1 when participants were aged 25 to 74. |
| Macalli et al., 2020 | Updated search | **Exposure measured in adulthood:** Perceived parental support assessed retrospectively in adulthood. |
| Markowitz, 2017 | Updated search | **Exposure measured into adulthood:** School connection measured at wave 2 (age 13-20) and spans adolescence into adulthood. |
| Metts et al., 2023 | Updated search | **Selected/high-risk sample:** Uses a selected/high-risk sample - oversampled for neuroticism. |
| Morris et al., 2021 | Original search | **Wrong study design:** This paper discusses two models based on existing child development literature as opposed to being an empirical longitudinal cohort study. |
| Narayan et al., 2017 | Original search | **Selected/high-risk sample:** The study sample were mothers reared in foster care and are not representative of the general population. |
| Narayan et al., 2018 | Original search | **Selected/high-risk sample:** The study used a sample of ethnically diverse, low-income pregnant women. |
| Narayan et al., 2021 | Original search | **Wrong study design:** The study was a review examining theory and evidence rather than an empirical longitudinal cohort study. |
| Nevarez-Brewster et al., 2022 | Original search | **Wrong outcome and selected/high-risk sample:** The outcome examined (sleep quality) was not within the scope of eligible outcomes and the study sample were pregnant women. |
| Novak and Fagan, 2022 | Updated search | **Outcome measured in childhood:** Outcome (offending behaviour) measured in adolescence rather than adulthood (>18 years). |
| Overbeek et al., 2004 | Original search | **Exposure measured in adulthood:** Exposure (recollections of parental bonds) assessed retrospectively in adulthood when participants were between 18 and 34 years old. |
| Park et al., 2021 | Updated search | **Wrong exposure:** Perceived parental control and warmth reflect parenting constructs/styles rather than discrete positive experiences. |
| Rasic et al., 2013 | Updated search | **Outcome measured in childhood:** Depression measured before age 18, while participants were still in school. |
| Rasmussen et al., 2021 | Updated search | **Wrong exposure:** No clearly defined positive childhood experiences as exposure(s). |
| Rodriguez et al., 2021 | Updated search | **Exposure measured in adulthood:** BCEs and positive human-animal interactions assessed via retrospective recall in adulthood. |
| Salazar et al., 2011 | Updated search | **Selected/high-risk sample:** Uses a cohort of youth exiting the public child welfare systems of three Midwestern states. |
| Schmid et al., 2011 | Original search | **Wrong exposure:** The exposures (two dimensions of mother-infant interaction including maternal stimulation and maternal responsiveness) were operationalised as continuous predictors and conceptualised as proximal risk factors rather than promotive/protective factors with higher levels conferring greater benefit. |
| Sheikh, 2018 | Updated search | **Exposure measured in adulthood:** Quantity and quality of social support measured in adulthood when participants were a mean age of 54.7 years. |
| Stavrakakis et al., 2012 | Original search | **Outcome measured in childhood:** The outcome of interest (depressive symptoms) was measured at three waves between age 10 and age 17 rather than in adulthood (age >18) |
| Ströhle et al., 2007 | Original search | **Exposure measured in adulthood:** Individuals were aged 14-24 years at the outset of the study, therefore some had exposure measured in adulthood rather than in childhood (age ≤ 18). |
| Upenieks et al., 2024 | Updated search | **Selected/high-risk sample:** Uses a trauma-enriched, non-general population cohort, which falls outside the eligibility criteria. |
| Wang et al., 2021 | Original search | **Selected/high-risk sample and outcome measured in childhood:** The study sample were socioeconomically disadvantaged adolescents and mental health outcomes (depression and anxiety) were measured at age 15 rather than in adulthood (age >18). |
| Whittle et al., 2014 | Original search | **Wrong outcome:** The outcome examined (brain structure measured by longitudinal structural MRI) was outside the scope of eligible mental health outcomes. |
| Williams et al., 2016 | Original search | **Wrong exposure:** The exposure examined (maternal-reported responses to infant crying) although related to the underlying PCE construct of ‘one safe caregiver’ does not adequately capture the presence of a positive caregiving experience, but rather may reflect variation in responsiveness that could be interpreted as the absence of adversity. |
| Wu et al., 2024 | Updated search | **Wrong outcome:** Insomnia falls outside the scope of eligible outcomes. |
| Zhang et al., 2021 | Original search | **Outcome measured in childhood:** The outcomes examined (depressive symptoms, oppositional defiant disorder (ODD), and conduct disorder (CD)) were assessed at wave 5 when participants were around 14 years of age rather than in adulthood (age >18). |
| Zhou, 2024 | Updated search | **Exposure measured in adulthood:** Childhood friendship experience assessed after age 18 - in adulthood via retrospective recall. |

**Table S5.** *Methodological quality assessment results*

| **Study** | **Study Design** | **JBI Checklist Items** | | | | | | | | | | | **Total**  **(% Yes)** | **Methodological Quality^§^** |
| --- | --- | --- | --- | --- | --- | --- | --- | --- | --- | --- | --- | --- | --- | --- |
|  |  | Q1 | Q2 | Q3 | Q4 | Q5 | Q6 | Q7 | Q8 | Q9 | Q10 | Q11 |  |  |
| Allen et al., 2024 | Prospective |  |  |  |  |  |  |  |  |  |  |  | 91% | High |
| Beyers and Seiffge-Krenke, 2007 | Prospective |  |  |  |  |  |  |  |  |  |  |  | 73% | Moderate |
| Brunet et al., 2013 | Prospective |  |  |  |  |  |  |  |  |  |  |  | 73% | Moderate |
| Cong et al., 2020 | Prospective |  |  |  |  |  |  |  |  |  |  |  | 82% | High |
| Crandall et al., 2020 | Prospective |  |  |  |  |  |  |  |  |  |  |  | 82% | High |
| Crush et al., 2018 | Prospective |  |  |  |  |  |  |  |  |  |  |  | 82% | High |
| da Silva et al., 2023 | Prospective |  |  |  |  |  |  |  |  |  |  |  | 82% | High |
| Glickman et al., 2021 | Prospective |  |  |  |  |  |  |  |  |  |  |  | 82% | High |
| Gondek et al., 2023 | Prospective |  |  |  |  |  |  |  |  |  |  |  | 82% | High |
| Howie et al., 2020 | Prospective |  |  |  |  |  |  |  |  |  |  |  | 82% | High |
| Kandola et al., 2020 | Prospective |  |  |  |  |  |  |  |  |  |  |  | 100% | High |
| Kemp et al., 2024 | Prospective |  |  |  |  |  |  |  |  |  |  |  | 73% | Moderate |
| Låftman et al., 2025 | Prospective |  |  |  |  |  |  |  |  |  |  |  | 82% | High |
| McKercher et al., 2014 | Prospective |  |  |  |  |  |  |  |  |  |  |  | 82% | High |
| Nyberg et al., 2019 | Prospective |  |  |  |  |  |  |  |  |  |  |  | 82% | High |
| Rollins and Crandall, 2021 | Prospective |  |  |  |  |  |  |  |  |  |  |  | 82% | High |
| Sagatun et al., 2007 | Prospective |  |  |  |  |  |  |  |  |  |  |  | 82% | High |
| Sormunen et al., 2017 | Prospective |  |  |  |  |  |  |  |  |  |  |  | 73% | Moderate |
| Steiner et al., 2019 | Prospective |  |  |  |  |  |  |  |  |  |  |  | 73% | Moderate |
| Upenieks and Thomas, 2021 | Prospective |  |  |  |  |  |  |  |  |  |  |  | 73% | Moderate |
| + = yes, - = no, ? = unclear ^§^Quality scores were categorised into three groups: Low: 0-4 ‘yes’ responses, Moderate: 5-8, and High: 9-11 | | | | | | | | | | | | | | |

**References**

Agerup, T., Lydersen, S., Wallander, J., & Sund, A. M. (2015). Associations Between Parental Attachment and Course of Depression Between Adolescence and Young Adulthood. *Child Psychiatry and Human Development*, *46*(4), 632–642. https://doi.org/10.1007/s10578-014-0506-y

Ahrens, K. R., DuBois, D. L., Richardson, L. P., Fan, M.-Y., & Lozano, P. (2008). Youth in foster care with adult mentors during adolescence have improved adult outcomes. *Pediatrics*, *121*(2), e246-52. https://doi.org/10.1542/peds.2007-0508

Allen, K.-A., Greenwood, C. J., Berger, E., Patlamazoglou, L., Reupert, A., Wurf, G., May, F., O’Connor, M., Sanson, A., Olsson, C. A., & Letcher, P. (2024). Adolescent School Belonging and Mental Health Outcomes in Young Adulthood: Findings from a Multi-wave Prospective Cohort Study. *School Mental Health*, *16*(1), 149–160. https://doi.org/10.1007/s12310-023-09626-6

Bang, H., Chang, M., & Kim, S. (2024). Team and individual sport participation, school belonging, and gender differences in adolescent depression. *Children and Youth Services Review*, *159*, 107517. https://doi.org/10.1016/j.childyouth.2024.107517

Banyard, V. L., Williams, L. M., Saunders, B. E., & Fitzgerald, M. M. (2008). The complexity of trauma types in the lives of women in families referred for family violence: Multiple mediators of mental health. *American Journal of Orthopsychiatry*, *78*(4), 394–404. https://doi.org/10.1037/a0014314

Baran, P., Pereira, D., Pocinho, M., Rodrigues, N., & Mamcarz, P. (2025). Protective factors against depression: the role of personality, spiritual fitness, and positive mental health in a two-wave longitudinal study of Polish adults. *Scientific Reports*, *15*(1), 45419. https://doi.org/10.1038/s41598-025-30165-4

Bates, E. J. S., Berny, L. M., Ganiban, J. M., Natsuaki, M. N., Neiderhiser, J. M., Shaw, D. S., & Leve, L. D. (2023). Examination of promotive and protective effects on early adolescent prosocial behavior through a bioecological lens. *Frontiers in Psychology*, *14*. https://doi.org/10.3389/fpsyg.2023.1280346

Bayar, Ö. (2025). Expanded Benevolent Childhood Experiences Scale (BCEs-20): Psychometric Properties and Comparison of BCEs-Original and BCEs-Revised. *Journal of Child and Family Studies*, *34*(11), 2976–2995. https://doi.org/10.1007/s10826-025-03169-x

Bethell, C., Jones, J., Gombojav, N., Linkenbach, J., & Sege, R. (2019). Positive Childhood Experiences and Adult Mental and Relational Health in a Statewide Sample. *JAMA Pediatrics*, *173*(11), e193007. https://doi.org/10.1001/jamapediatrics.2019.3007

Beyers, W., & Seiffge-Krenke, I. (2007). Are friends and romantic partners the “best medicine”? How the quality of other close relations mediates the impact of changing family relationships on adjustment. *International Journal of Behavioral Development*, *31*(6), 559–568. https://doi.org/10.1177/0165025407080583

Brieant, A., & Burt, K. B. (2025). The protective role of community cohesion across rural and urban contexts: implications for youth mental health. *Child and Adolescent Mental Health*, *30*(2), 140–148. https://doi.org/10.1111/camh.12764

Bromley, K., Sacks, D. D., Boyes, A., Driver, C., & Hermens, D. F. (2024). Health enhancing behaviors in early adolescence: an investigation of nutrition, sleep, physical activity, mindfulness and social connectedness and their association with psychological distress and wellbeing. *Frontiers in Psychiatry*, *15*. https://doi.org/10.3389/fpsyt.2024.1413268

Brown, C., Douthwaite, A., Donnelly, M., & Olaniyan, Y. D. (2025). Resilience Through Belonging: Schools’ Role in Promoting the Mental Health and Well-Being of Children and Young People. *Behavioral Sciences*, *15*(10), 1421. https://doi.org/10.3390/bs15101421

Brunet, J., Sabiston, C. M., Chaiton, M., Barnett, T. A., O’Loughlin, E., Low, N. C. P., & O’Loughlin, J. L. (2013). The association between past and current physical activity and depressive symptoms in young adults: a 10-year prospective study. *Annals of Epidemiology*, *23*(1), 25–30. https://doi.org/10.1016/j.annepidem.2012.10.006

Buchanan, M., Newton-Howes, G., Cunningham, R., McLeod, G. F. H., & Boden, J. M. (2024). The role of social support in reducing the long-term burden of cumulative childhood adversity on adulthood internalising disorder. *Social Psychiatry and Psychiatric Epidemiology*, *59*(12), 2165–2175. https://doi.org/10.1007/s00127-024-02674-6

Cahill, S., Hager, R., & Chandola, T. (2022). The validity of the residuals approach to measuring resilience to adverse childhood experiences. *Child and Adolescent Psychiatry and Mental Health*, *16*(1), 18. https://doi.org/10.1186/s13034-022-00449-y

Cammack, A. L., & Suglia, S. F. (2023). Mentorship in adolescence and subsequent depression and adiposity among child maltreatment survivors in a United States nationally representative sample. *Preventive Medicine*, *166*, 107339. https://doi.org/10.1016/j.ypmed.2022.107339

Cárdenas, E. F., Kujawa, A., & Humphreys, K. L. (2022). Benevolent Childhood Experiences and Childhood Maltreatment History: Examining Their Roles in Depressive Symptoms Across the Peripartum Period. *Adversity and Resilience Science*, *3*(2), 169–179. https://doi.org/10.1007/s42844-022-00062-0

Chen, E., Yu, T., Siliezar, R., Drage, J. N., Dezil, J., Miller, G. E., & Brody, G. H. (2020). Evidence for skin-deep resilience using a co-twin control design: Effects on low-grade inflammation in a longitudinal study of youth. *Brain, Behavior, and Immunity*, *88*, 661–667. https://doi.org/10.1016/j.bbi.2020.04.070

Chen, P., & Harris, K. M. (2019). Association of Positive Family Relationships With Mental Health Trajectories From Adolescence to Midlife. *JAMA Pediatrics*, *173*(12), e193336. https://doi.org/10.1001/jamapediatrics.2019.3336

Chen, Y., & VanderWeele, T. J. (2018). Associations of Religious Upbringing With Subsequent Health and Well-Being From Adolescence to Young Adulthood: An Outcome-Wide Analysis. *American Journal of Epidemiology*, *187*(11), 2355–2364. https://doi.org/10.1093/aje/kwy142

Chung, E. K., Mathew, L., Elo, I. T., Coyne, J. C., & Culhane, J. F. (2008). Depressive Symptoms in Disadvantaged Women Receiving Prenatal Care: The Influence of Adverse and Positive Childhood Experiences. *Ambulatory Pediatrics*, *8*(2), 109–116. https://doi.org/10.1016/j.ambp.2007.12.003

Clements-Nolle, K., & Waddington, R. (2019). Adverse Childhood Experiences and Psychological Distress in Juvenile Offenders: The Protective Influence of Resilience and Youth Assets. *Journal of Adolescent Health*, *64*(1), 49–55. https://doi.org/10.1016/j.jadohealth.2018.09.025

Cong, X., Hosler, A. S., Tracy, M., & Appleton, A. A. (2020). The relationship between parental involvement in childhood and depression in early adulthood. *Journal of Affective Disorders*, *273*, 173–182. https://doi.org/10.1016/j.jad.2020.03.108

Corallo, K. L., Carr, C. P., Lavner, J. A., Koss, K. J., & Ehrlich, K. B. (2023). The protective role of parental vigilance in the link between risky childhood environments and health. *Social Science & Medicine*, *317*, 115593. https://doi.org/10.1016/j.socscimed.2022.115593

Crandall, A., Broadbent, E., Stanfill, M., Magnusson, B. M., Novilla, M. L. B., Hanson, C. L., & Barnes, M. D. (2020). The influence of adverse and advantageous childhood experiences during adolescence on young adult health. *Child Abuse & Neglect*, *108*(104644), 104644. https://doi.org/10.1016/j.chiabu.2020.104644

Crandall, A., Miller, J. R., Cheung, A., Novilla, L. K., Glade, R., Novilla, M. L. B., Magnusson, B. M., Leavitt, B. L., Barnes, M. D., & Hanson, C. L. (2019). ACEs and counter-ACEs: How positive and negative childhood experiences influence adult health. *Child Abuse & Neglect*, *96*, 104089. https://doi.org/10.1016/j.chiabu.2019.104089

Crush, E., Arseneault, L., Moffitt, T. E., Danese, A., Caspi, A., Jaffee, S. R., Matthews, T., & Fisher, H. L. (2018). Protective factors for psychotic experiences amongst adolescents exposed to multiple forms of victimization. *Journal of Psychiatric Research*, *104*, 32–38. https://doi.org/10.1016/j.jpsychires.2018.06.011

da Silva, B. G. C., Menezes, A. M. B., Gonçalves, H., Alves, C. P. de L., Delpino, F. M., Martins, R. C., & Wehrmeister, F. C. (2023). Physical Activity During Adolescence and Mental Health in Early Adulthood: Findings From the 1993 Pelotas Birth Cohort Study. *Journal of Physical Activity and Health*, *20*(9), 860–867. https://doi.org/10.1123/jpah.2022-0658

Delawalla, M. (2019). *A Concurrent and Longitudinal Analysis of Teacher Support, Stressful Life Events, and Depressive Symptoms in Early Adolescence* [Master of Public Health Thesis]. University of Washington.

Dibben, C., Playford, C., & Mitchell, R. (2017). Be(ing) prepared: Guide and Scout participation, childhood social position and mental health at age 50—a prospective birth cohort study. *Journal of Epidemiology and Community Health*, *71*(3), 275–281. https://doi.org/10.1136/jech-2016-207898

Easterlin, M. C., Chung, P. J., Leng, M., & Dudovitz, R. (2019). Association of Team Sports Participation With Long-term Mental Health Outcomes Among Individuals Exposed to Adverse Childhood Experiences. *JAMA Pediatrics*, *173*(7), 681. https://doi.org/10.1001/jamapediatrics.2019.1212

Eshel, N., Daelmans, B., Cabral de Mello, M., & Martines, J. (2006). Responsive parenting: interventions and outcomes. *Bulletin of the World Health Organization*, *84*(12), 991–998. https://doi.org/10.2471/BLT.06.030163

Fang, S., Fosco, G. M., & Feinberg, M. E. (2024). Parental warmth and young adult depression: A comparison of enduring effects and revisionist models. *Development and Psychopathology*, *36*(4), 1849–1862. https://doi.org/10.1017/S0954579423001207

Fitzgerald, M., & Gallus, K. (2020). Emotional support as a mechanism linking childhood maltreatment and adult’s depressive and social anxiety symptoms. *Child Abuse & Neglect*, *108*, 104645. https://doi.org/10.1016/j.chiabu.2020.104645

Fitzpatrick, M. M., Anderson, A. M., Browning, C., & Ford, J. L. (2024). Relationship Between Family and Friend Support and Psychological Distress in Adolescents. *Journal of Pediatric Health Care*, *38*(6), 804–811. https://doi.org/10.1016/j.pedhc.2024.06.016

Galera, C., Navarro, M. C., Galesne, C., Retuerto, N., Bentivegna, F., & Flouri, E. (2025). Neighborhood green space and psychological distress: a longitudinal study of socioeconomic disparities in mental health outcomes. *Environment International*, *204*, 109799. https://doi.org/10.1016/j.envint.2025.109799

Ganjeh, P., Hagmayer, Y., Meyer, T., Kuhnert, R., Ravens-Sieberer, U., von Steinbuechel, N., Rothenberger, A., & Becker, A. (2024). Physical activity and psychopathology: are long-term developmental trajectories of physical activity in children and adolescents associated with trajectories of general mental health problems and of attention-deficit hyperactivity (ADHD) symptoms? *European Child & Adolescent Psychiatry*, *33*(9), 3067–3078. https://doi.org/10.1007/s00787-023-02352-z

Garnsey, C. L., Park, C. L., Magin, Z. E., Russell, B. S., & Milan, S. (2026). Self-compassion as a shield? Investigating self-compassion’s prospective role in buffering the effects of traumatic and negative life events. *Anxiety, Stress, & Coping*, *39*(1), 73–87. https://doi.org/10.1080/10615806.2025.2539169

Glickman, E. A., Choi, K. W., Lussier, A. A., Smith, B. J., & Dunn, E. C. (2021). Childhood Emotional Neglect and Adolescent Depression: Assessing the Protective Role of Peer Social Support in a Longitudinal Birth Cohort. *Frontiers in Psychiatry*, *12*. https://doi.org/10.3389/fpsyt.2021.681176

Godoy-Casasbuenas, N., Gómez-Restrepo, C., Rodríguez Malagón, N., Diez-Canseco, F., Ramirez-Meneses, D., Flores, S., Brusco, L. I., Carbonetti, F. L., Olivar, N., Bird, V., Kirkbride, J. B., Priebe, S., & Uribe-Restrepo, J. M. (2025). The Role of Perceived Social Support in the Recovery of Depressive and Anxiety Symptoms in Adolescents and Young Adults: A 24-Month Longitudinal Study in Three South American Cities. *Revista Colombiana de Psiquiatría*, *54*, 3–14. https://doi.org/10.1016/j.rcp.2025.06.002

Gondek, D., Feder, G., Howe, L. D., Gilbert, R., Howarth, E., Deighton, J., & Lacey, R. E. (2023). Factors mitigating the harmful effects of intimate partner violence on adolescents’ depressive symptoms—A longitudinal birth cohort study. *JCPP Advances*, *3*(1). https://doi.org/10.1002/jcv2.12134

Goodrum, N. M., Smith, D. W., Hanson, R. F., Moreland, A. D., Saunders, B. E., & Kilpatrick, D. G. (2020). Longitudinal Relations among Adolescent Risk Behavior, Family Cohesion, Violence Exposure, and Mental Health in a National Sample. *Journal of Abnormal Child Psychology*, *48*(11), 1455–1469. https://doi.org/10.1007/s10802-020-00691-y

Gunnarsdóttir, H., Hensing, G., & Hammarström, A. (2021). Poor school connectedness in adolescence and adulthood depressiveness: a longitudinal theory-driven study from the Northern Sweden Cohort. *European Journal of Public Health*, *31*(4), 797–802. https://doi.org/10.1093/eurpub/ckab027

Hardi, F. A., Keding, T. J., Holt-Gosselin, B., Goldfarb, E. V., & Gee, D. G. (2026). Childhood environment clusters reveal heterogeneous associations between large-scale brain networks and youth mental health symptoms. *Psychological Medicine*, *56*, e134. https://doi.org/10.1017/S0033291726104115

Hays-Grudo, J., Morris, A. S., Beasley, L., Ciciolla, L., Shreffler, K., & Croff, J. (2021). Integrating and synthesizing adversity and resilience knowledge and action: The ICARE model. *American Psychologist*, *76*(2), 203–215. https://doi.org/10.1037/amp0000766

Herath, B., & Zhang, X. (2025). Everyday Discrimination in Young Adulthood and Depressive Symptoms at Early Midlife: The Moderating Role of Parent–Child Relationships. *International Journal of Environmental Research and Public Health*, *22*(9), 1323. https://doi.org/10.3390/ijerph22091323

Herman, K. A., Hautala, D. S., Aulandez, K. M. W., & Walls, M. L. (2024). The resounding influence of benevolent childhood experiences. *Transcultural Psychiatry*, *61*(3), 339–350. https://doi.org/10.1177/13634615231192006

Herrenkohl, T. I., Jung, H., Klika, J. B., Mason, W. A., Brown, E. C., Leeb, R. T., & Herrenkohl, R. C. (2016). Mediating and moderating effects of social support in the study of child abuse and adult physical and mental health. *American Journal of Orthopsychiatry*, *86*(5), 573–583. https://doi.org/10.1037/ort0000136

Howie, E. K., McVeigh, J. A., Smith, A. J., Zabatiero, J., Bucks, R. S., Mori, T. A., Beilin, L. J., & Straker, L. M. (2020). Physical activity trajectories from childhood to late adolescence and their implications for health in young adulthood. *Preventive Medicine*, *139*, 106224. https://doi.org/10.1016/j.ypmed.2020.106224

Huang, C. X., Halfon, N., Sastry, N., Chung, P. J., & Schickedanz, A. (2023). Positive Childhood Experiences and Adult Health Outcomes. *Pediatrics*, *152*(1). https://doi.org/10.1542/peds.2022-060951

Hurd, N. M., & Zimmerman, M. A. (2014). An Analysis of Natural Mentoring Relationship Profiles and Associations with Mentees’ Mental Health: Considering Links via Support from Important Others. *American Journal of Community Psychology*, *53*(1–2), 25–36. https://doi.org/10.1007/s10464-013-9598-y

Hurd, N., & Zimmerman, M. (2010). Natural Mentors, Mental Health, and Risk Behaviors: A Longitudinal Analysis of African American Adolescents Transitioning into Adulthood. *American Journal of Community Psychology*, *46*(1–2), 36–48. https://doi.org/10.1007/s10464-010-9325-x

Huynh, M. P., Choi, Y., & Ro, A. (2025). The role of friend support on the relationship between family stressors and mental health of Korean and Filipino American youth. *Asian American Journal of Psychology*, *16*(4), 397–408. https://doi.org/10.1037/aap0000388

Iyer, P., Parmar, D., Ganson, K. T., Tabler, J., Soleimanpour, S., & Nagata, J. M. (2023). Investigating Asian American Adolescents’ Resiliency Factors and Young Adult Mental Health Outcomes at 14-year Follow-up: A Nationally Representative Prospective Cohort Study. *Journal of Immigrant and Minority Health*, *25*(1), 75–85. https://doi.org/10.1007/s10903-022-01373-1

Jacka, F. N., Pasco, J. A., Williams, L. J., Leslie, E. R., Dodd, S., Nicholson, G. C., Kotowicz, M. A., & Berk, M. (2011). Lower levels of physical activity in childhood associated with adult depression. *Journal of Science and Medicine in Sport*, *14*(3), 222–226. https://doi.org/10.1016/j.jsams.2010.10.458

Jerstad, S. J., Boutelle, K. N., Ness, K. K., & Stice, E. (2010). Prospective reciprocal relations between physical activity and depression in female adolescents. *Journal of Consulting and Clinical Psychology*, *78*(2), 268–272. https://doi.org/10.1037/a0018793

Kandola, A., Lewis, G., Osborn, D. P. J., Stubbs, B., & Hayes, J. F. (2020). Depressive symptoms and objectively measured physical activity and sedentary behaviour throughout adolescence: a prospective cohort study. *The Lancet Psychiatry*, *7*(3), 262–271. https://doi.org/10.1016/S2215-0366(20)30034-1

Kemp, L., Elcombe, E., Blythe, S., Grace, R., Donohoe, K., & Sege, R. (2024). The Impact of Positive and Adverse Experiences in Adolescence on Health and Wellbeing Outcomes in Early Adulthood. *International Journal of Environmental Research and Public Health*, *21*(9), 1147. https://doi.org/10.3390/ijerph21091147

Kendler, K. S., Myers, J., & Prescott, C. A. (2005). Sex Differences in the Relationship Between Social Support and Risk for Major Depression: A Longitudinal Study of Opposite-Sex Twin Pairs. *American Journal of Psychiatry*, *162*(2), 250–256. https://doi.org/10.1176/appi.ajp.162.2.250

Kent, B. V., & Bradshaw, M. (2021). Adolescent Context and Depressive Symptom Trajectories in a National Sample: Ages 13 to 34. *International Journal of Mental Health and Addiction*, *19*(5), 1468–1484. https://doi.org/10.1007/s11469-020-00236-w

Kim, E. S., Wilkinson, R., Case, B. W., Cowden, R. G., Okuzono, S. S., & VanderWeele, T. J. (2024). Connected communities: Perceived neighborhood social cohesion during adolescence and subsequent health and well‐being in young adulthood—An outcome‐wide longitudinal approach. *Journal of Community Psychology*, *52*(6), 774–791. https://doi.org/10.1002/jcop.23130

Kim, Y., Lee, H., & Park, A. (2022). Patterns of adverse childhood experiences and depressive symptoms: self-esteem as a mediating mechanism. *Social Psychiatry and Psychiatric Epidemiology*, *57*(2), 331–341. https://doi.org/10.1007/s00127-021-02129-2

Kingsbury, M., Clayborne, Z., Colman, I., & Kirkbride, J. B. (2020). The protective effect of neighbourhood social cohesion on adolescent mental health following stressful life events. *Psychological Medicine*, *50*(8), 1292–1299. https://doi.org/10.1017/S0033291719001235

Knappe, S., Beesdo, K., Fehm, L., Höfler, M., Lieb, R., & Wittchen, H.-U. (2009). Do parental psychopathology and unfavorable family environment predict the persistence of social phobia? *Journal of Anxiety Disorders*, *23*(7), 986–994. https://doi.org/10.1016/j.janxdis.2009.06.010

Knappe, S., Beesdo, K., Fehm, L., Lieb, R., & Wittchen, H.-U. (2009). Associations of familial risk factors with social fears and social phobia: evidence for the continuum hypothesis in social anxiety disorder? *Journal of Neural Transmission*, *116*(6), 639–648. https://doi.org/10.1007/s00702-008-0118-4

Knappe, S., Lieb, R., Beesdo, K., Fehm, L., Ping Low, N. C., Gloster, A. T., & Wittchen, H.-U. (2009). The role of parental psychopathology and family environment for social phobia in the first three decades of life. *Depression and Anxiety*, *26*(4), 363–370. https://doi.org/10.1002/da.20527

König, M., Smith, A. J., Moreno-López, L., Davidson, E., Dauvermann, M., Orellana, S., McCormick, E. M., Peris, T. S., Kaser, M., Ioannidis, K., & van Harmelen, A.-L. (2025). Friendship buffering effects on mental health symptoms before and during the COVID-19 pandemic: A UK longitudinal study of young people with childhood adversity. *Development and Psychopathology*, *37*(5), 2386–2401. https://doi.org/10.1017/S0954579424001986

Korhonen, T., Kujala, U. M., Rose, R. J., & Kaprio, J. (2009). Physical Activity in Adolescence as a Predictor of Alcohol and Illicit Drug Use in Early Adulthood: A Longitudinal Population-Based Twin Study. *Twin Research and Human Genetics*, *12*(3), 261–268. https://doi.org/10.1375/twin.12.3.261

Kosterman, R., Mason, W. A., Haggerty, K. P., Hawkins, J. D., Spoth, R., & Redmond, C. (2011). Positive Childhood Experiences and Positive Adult Functioning: Prosocial Continuity and the Role of Adolescent Substance Use. *Journal of Adolescent Health*, *49*(2), 180–186. https://doi.org/10.1016/j.jadohealth.2010.11.244

Låftman, S. B., Lundin, A., & Östberg, V. (2025). Perceived social support and symptoms of depression and anxiety in emerging adulthood: A Swedish prospective cohort study. *Scandinavian Journal of Public Health*, *53*(8), 854–862. https://doi.org/10.1177/14034948241290927

Latham, R. M., Arseneault, L., Alexandrescu, B., Baldoza, S., Carter, A., Moffitt, T. E., Newbury, J. B., & Fisher, H. L. (2022). Violent experiences and neighbourhoods during adolescence: understanding and mitigating the association with mental health at the transition to adulthood in a longitudinal cohort study. *Social Psychiatry and Psychiatric Epidemiology*, *57*(12), 2379–2391. https://doi.org/10.1007/s00127-022-02343-6

Lewis, K. N., & McKelvey, L. M. (2025). Positive childhood experiences support emotional and behavioral health in middle childhood: Longitudinal mediation of adverse childhood experiences. *Child Abuse & Neglect*, *163*, 107320. https://doi.org/10.1016/j.chiabu.2025.107320

Lim, Y. (2025). Longitudinal association between consecutive moderate-to-vigorous physical activity and the risk of depression among depressed and non-depressed participants: A nationally representative cohort study. *Journal of Affective Disorders*, *381*, 475–483. https://doi.org/10.1016/j.jad.2025.03.048

Luby, J. L., Tillman, R., & Barch, D. M. (2019). Association of Timing of Adverse Childhood Experiences and Caregiver Support With Regionally Specific Brain Development in Adolescents. *JAMA Network Open*, *2*(9), e1911426. https://doi.org/10.1001/jamanetworkopen.2019.11426

Lundgren, O., Tigerstrand, H., Lebena, A., Löf, M., & Ludvigsson, J. (2025). Impact of physical activity on the incidence of psychiatric conditions during childhood: a longitudinal Swedish birth cohort study. *British Journal of Sports Medicine*, *59*(14), 1001–1009. https://doi.org/10.1136/bjsports-2024-108148

Lyu, J., & Agrigoroaei, S. (2017). Childhood Misfortune and Adult Health in a National Study. *The International Journal of Aging and Human Development*, *84*(3), 213–230. https://doi.org/10.1177/0091415016669147

Macalli, M., Côté, S., & Tzourio, C. (2020). Perceived parental support in childhood and adolescence as a tool for mental health screening in students: A longitudinal study in the i-Share cohort. *Journal of Affective Disorders*, *266*, 512–519. https://doi.org/10.1016/j.jad.2020.02.009

Markowitz, A. J. (2017). Associations Between School Connection and Depressive Symptoms From Adolescence Through Early Adulthood: Moderation by Early Adversity. *Journal of Research on Adolescence*, *27*(2), 298–311. https://doi.org/10.1111/jora.12275

Maurer, J., Meyrose, A.-K., Kaman, A., Mauz, E., Ravens-Sieberer, U., & Reiss, F. (2025). Socioeconomic Status, Protective Factors, and Mental Health Problems in Transition from Adolescence to Emerging Adulthood: Results of the Longitudinal BELLA Study. *Child Psychiatry & Human Development*, *56*(3), 649–660. https://doi.org/10.1007/s10578-023-01582-1

McKercher, C., Sanderson, K., Schmidt, M. D., Otahal, P., Patton, G. C., Dwyer, T., & Venn, A. J. (2014). Physical activity patterns and risk of depression in young adulthood: a 20-year cohort study since childhood. *Social Psychiatry and Psychiatric Epidemiology*, *49*(11), 1823–1834. https://doi.org/10.1007/s00127-014-0863-7

McPhie, M. L., & Rawana, J. S. (2015). The effect of physical activity on depression in adolescence and emerging adulthood: A growth‐curve analysis. *Journal of Adolescence*, *40*(1), 83–92. https://doi.org/10.1016/j.adolescence.2015.01.008

Metts, A. V., Yarrington, J. S., Zinbarg, R., Hammen, C., Mineka, S., Enders, C., & Craske, M. G. (2023). Early-life adversity and risk for depression and anxiety: The role of interpersonal support. *Development and Psychopathology*, *35*(2), 863–875. https://doi.org/10.1017/S0954579422000116

Miller, K., Morley, C., Fraser, B. J., Gall, S. L., & Cleland, V. (2024). Types of leisure-time physical activity participation in childhood and adolescence, and physical activity behaviours and health outcomes in adulthood: a systematic review. *BMC Public Health*, *24*(1), 1789. https://doi.org/10.1186/s12889-024-19050-3

Mondi, C. F., Reynolds, A. J., & Richardson, B. A. (2020). Early Childhood Education and Adult Depression: An Attrition Reanalysis With Inverse Propensity Score Weighting. *Evaluation Review*, *44*(5–6), 379–409. https://doi.org/10.1177/0193841X20976527

Morris, A. S., Hays-Grudo, J., Kerr, K. L., & Beasley, L. O. (2021). The heart of the matter: Developing the whole child through community resources and caregiver relationships. *Development and Psychopathology*, *33*(2), 533–544. https://doi.org/10.1017/S0954579420001595

Narayan, A. J., Ippen, C. G., Harris, W. W., & Lieberman, A. F. (2017). Assessing angels in the nursery: A pilot study of childhood memories of benevolent caregiving as protective influences. *Infant Mental Health Journal*, *38*(4), 461–474. https://doi.org/10.1002/imhj.21653

Narayan, A. J., Lieberman, A. F., & Masten, A. S. (2021). Intergenerational transmission and prevention of adverse childhood experiences (ACEs). *Clinical Psychology Review*, *85*, 101997. https://doi.org/10.1016/j.cpr.2021.101997

Narayan, A. J., Rivera, L. M., Bernstein, R. E., Harris, W. W., & Lieberman, A. F. (2018). Positive childhood experiences predict less psychopathology and stress in pregnant women with childhood adversity: A pilot study of the benevolent childhood experiences (BCEs) scale. *Child Abuse & Neglect*, *78*, 19–30. https://doi.org/10.1016/j.chiabu.2017.09.022

Nevarez-Brewster, M., Aran, Ö., Narayan, A. J., Harrall, K. K., Brown, S. M., Hankin, B. L., & Davis, E. P. (2022). Adverse and Benevolent Childhood Experiences Predict Prenatal Sleep Quality. *Adversity and Resilience Science*, *3*(4), 391–402. https://doi.org/10.1007/s42844-022-00070-0

Novak, A., & Fagan, A. A. (2022). The conditioning effects of positive experiences on the ACEs-offending relationship in adolescence. *Child Abuse & Neglect*, *134*, 105915. https://doi.org/10.1016/j.chiabu.2022.105915

Nyberg, A., Rajaleid, K., Westerlund, H., & Hammarström, A. (2019). Does social and professional establishment at age 30 mediate the association between school connectedness and family climate at age 16 and mental health symptoms at age 43? *Journal of Affective Disorders*, *246*, 52–61. https://doi.org/10.1016/j.jad.2018.12.027

O’Reilly, L., Elam, K. K., Quinn, P. D., Adams, S., Chirica, M. G., Klonsky, E. D., Pettersson, E., Lundström, S., Larsson, H., Lichtenstein, P., & D’Onofrio, B. (2022). Examining protective factors for substance use problems and self-harm behavior during adolescence: A longitudinal co-twin control study. *Development and Psychopathology*, *34*(5), 1781–1802. https://doi.org/10.1017/S0954579422000724

Overbeek, G., Vollebergh, W., Meeus, W., de Graaf, R., & Engels, R. C. M. E. (2004). Young adults’ recollections of parental bonds. *Social Psychiatry and Psychiatric Epidemiology*, *39*(9), 703–710. https://doi.org/10.1007/s00127-004-0806-9

Park, S.-Y., Lee, J., & Cheah, C. S. L. (2021). The long-term effects of perceived parental control and warmth on self-esteem and depressive symptoms among Asian American youth. *Children and Youth Services Review*, *126*, 105999. https://doi.org/10.1016/j.childyouth.2021.105999

Patten, S. B., Williams, J. V. A., Lavorato, D. H., & Eliasziw, M. (2009). A longitudinal community study of major depression and physical activity. *General Hospital Psychiatry*, *31*(6), 571–575. https://doi.org/10.1016/j.genhosppsych.2009.08.001

Pérez Rodríguez, S., Layrón Folgado, J. E., Guillén Botella, V., & Marco Salvador, J. H. (2024). Meaning in life mediates the association between depressive symptoms and future frequency of suicidal ideation in Spanish university students: A longitudinal study. *Suicide and Life-Threatening Behavior*, *54*(2), 286–295. https://doi.org/10.1111/sltb.13040

Ranøyen, I., Wallander, J. L., Lydersen, S., Thomsen, P. H., & Jozefiak, T. (2025). Promotive factors associated with reduced anxiety and depression across three years in a prospective clinical cohort of adolescents: Examining compensatory and protective models of resilience. *Development and Psychopathology*, *37*(4), 1903–1918. https://doi.org/10.1017/S0954579424001469

Rasic, D., Asbridge, M., Kisely, S., & Langille, D. (2013). Longitudinal Associations of Importance of Religion and Frequency of Service Attendance with Depression Risk among Adolescents in Nova Scotia. *The Canadian Journal of Psychiatry*, *58*(5), 291–299. https://doi.org/10.1177/070674371305800507

Rasmussen, Z. A., Shah, W. H., Hansen, C. L., Azam, S. I., Hussain, E., Schaefer, B. A., Zhong, N., Jamison, A. F., Ahmed, K., & McCormick, B. J. J. (2021). Examining the relationships between early childhood experiences and adolescent and young adult health status in a resource-limited population: A cohort study. *PLOS Medicine*, *18*(9), e1003745. https://doi.org/10.1371/journal.pmed.1003745

Ren, M., Song, J., Zhou, C., Hou, J., Huang, H., & Li, L. (2024). Effects of Family Environment on Depressive Symptoms in Postgraduate Students: Longitudinal Moderating Effect of Family Support and Mediating Effect of Psychological Resilience. *Depression and Anxiety*, *2024*(1). https://doi.org/10.1155/da/3867823

Rodriguez, K. E., McDonald, S. E., & Brown, S. M. (2021). Relationships among Early Adversity, Positive Human and Animal Interactions, and Mental Health in Young Adults. *Behavioral Sciences*, *11*(12), 178. https://doi.org/10.3390/bs11120178

Rollins, E. M., & Crandall, A. (2021). Self-Regulation and Shame as Mediators Between Childhood Experiences and Young Adult Health. *Frontiers in Psychiatry*, *12*. https://doi.org/10.3389/fpsyt.2021.649911

Rueger, S. Y., & George, R. (2017). Indirect Effects of Attributional Style for Positive Events on Depressive Symptoms Through Self-Esteem During Early Adolescence. *Journal of Youth and Adolescence*, *46*(4), 701–708. https://doi.org/10.1007/s10964-016-0530-2

Sagatun, A., Søgaard, A. J., Bjertness, E., Selmer, R., & Heyerdahl, S. (2007). The association between weekly hours of physical activity and mental health: A three-year follow-up study of 15–16-year-old students in the city of Oslo, Norway. *BMC Public Health*, *7*(1), 155. https://doi.org/10.1186/1471-2458-7-155

Sahib, A., Chen, J., Reynolds, K. J., & Cárdenas, D. (2025). The curative effect of schools: A longitudinal study of the impact of school climate, school identification, and resilience on adolescent mental health. *School Psychology*, *40*(1), 13–23. https://doi.org/10.1037/spq0000571

Salazar, A. M., Keller, T. E., & Courtney, M. E. (2011). Understanding Social Support’s Role in the Relationship Between Maltreatment and Depression in Youth With Foster Care Experience. *Child Maltreatment*, *16*(2), 102–113. https://doi.org/10.1177/1077559511402985

Salvatore, J. E., Collins, W. A., & Simpson, J. A. (2011). Moderating Processes in the Link between Early Caregiving and Adult Individual and Romantic Functioning: The Distinctive Contributions of Early Adult Romantic Relationships. In *Dissertation*.

Schmid, B., Blomeyer, D., Buchmann, A. F., Trautmann-Villalba, P., Zimmermann, U. S., Schmidt, M. H., Esser, G., Banaschewski, T., & Laucht, M. (2011). Quality of early mother–child interaction associated with depressive psychopathology in the offspring: A prospective study from infancy to adulthood. *Journal of Psychiatric Research*, *45*(10), 1387–1394. https://doi.org/10.1016/j.jpsychires.2011.05.010

Sheikh, M. A. (2018). The potential protective effect of friendship on the association between childhood adversity and psychological distress in adulthood: A retrospective, preliminary, three-wave population-based study. *Journal of Affective Disorders*, *226*, 21–27. https://doi.org/10.1016/j.jad.2017.09.015

Shoshani, A. (2024). Longitudinal changes in children’s and adolescents’ mental health and well-being and associated protective factors during the COVID-19 pandemic. *Psychological Trauma: Theory, Research, Practice, and Policy*, *16*(7), 1158–1168. https://doi.org/10.1037/tra0001556

Skodol, A. E., Bender, D. S., Pagano, M. E., Shea, M. T., Yen, S., Sanislow, C. A., Grilo, C. M., Daversa, M. T., Stout, R. L., Zanarini, M. C., McGlashan, T. H., & Gunderson, J. G. (2007). Positive childhood experiences: resilience and recovery from personality disorder in early adulthood. *The Journal of Clinical Psychiatry*, *68*(7), 1102–1108. https://doi.org/10.4088/JCP.v68n0719

Sormunen, E., Saarinen, M. M., Salokangas, R. K. R., Telama, R., Hutri-Kähönen, N., Tammelin, T., Viikari, J., Raitakari, O., & Hietala, J. (2017). Effects of childhood and adolescence physical activity patterns on psychosis risk—a general population cohort study. *Npj Schizophrenia*, *3*(1), 5. https://doi.org/10.1038/s41537-016-0007-z

Stavrakakis, N., De Jonge, P., Ormel, J., & Oldehinkel, A. J. (2012). Bidirectional prospective associations between physical activity and depressive symptoms. the TRAILS study. *Journal of Adolescent Health*, *50*(5), 503–508. https://doi.org/10.1016/j.jadohealth.2011.09.004

Stavrakakis, N., Roest, A. M., Verhulst, F., Ormel, J., de Jonge, P., & Oldehinkel, A. J. (2013). Physical activity and onset of depression in adolescents: A prospective study in the general population cohort TRAILS. *Journal of Psychiatric Research*, *47*(10), 1304–1308. https://doi.org/10.1016/j.jpsychires.2013.06.005

Steiner, R. J., Sheremenko, G., Lesesne, C., Dittus, P. J., Sieving, R. E., & Ethier, K. A. (2019). Adolescent Connectedness and Adult Health Outcomes. *Pediatrics*, *144*(1). https://doi.org/10.1542/peds.2018-3766

Stock, S., Lacey, R., Fisher, H., & Baldwin, J. (2023). OP147 Can a warm and supportive adult protect against mental health problems amongst children with experience of adversity? A twin difference study. *SSM Annual Scientific Meeting*, A133.1-A133. https://doi.org/10.1136/jech-2023-SSMabstracts.277

Ströhle, A., Höfler, M., Pfister, H., Müller, A.-G., Hoyer, J., Wittchen, H.-U., & Lieb, R. (2007). Physical activity and prevalence and incidence of mental disorders in adolescents and young adults. *Psychological Medicine*, *37*(11), 1657–1666. https://doi.org/10.1017/S003329170700089X

Suetani, S., Mamun, A., Williams, G. M., Najman, J. M., McGrath, J. J., & Scott, J. G. (2017). Longitudinal association between physical activity engagement during adolescence and mental health outcomes in young adults: A 21-year birth cohort study. *Journal of Psychiatric Research*, *94*, 116–123. https://doi.org/10.1016/j.jpsychires.2017.06.013

Suetani, S., Mamun, A., Williams, G., Najman, J., McGrath, J., & Scott, J. (2018). RANZCP Abstracts: Longitudinal association between physical activity engagement during adolescence and mental health outcomes in young adults: A 21-year birth cohort study. *Australian & New Zealand Journal of Psychiatry*, *52*(1_suppl), 3–156. https://doi.org/10.1177/0004867418764980

Svensson, M., Brundin, L., Erhardt, S., Hållmarker, U., James, S., & Deierborg, T. (2021). Physical Activity Is Associated With Lower Long-Term Incidence of Anxiety in a Population-Based, Large-Scale Study. *Frontiers in Psychiatry*, *12*. https://doi.org/10.3389/fpsyt.2021.714014

Tietbohl-Santos, B., Montezano, B. B., de Azevedo Cardoso, T., Mondin, T. C., Moreira, F. P., Souza, L. D. de M., da Silva, R. A., Kapczinski, F., Jansen, K., & Passos, I. C. (2025). Exploring protective factors in a high-risk subsample: the pivotal role of paternal support in preventing depression in a cohort of young adults. *Trends in Psychiatry and Psychotherapy*, *47*. https://doi.org/10.47626/2237-6089-2024-0804

Tort-Carrera, A., Elhorst, J. P., & Bijwaard, G. E. (2026). The Impact of Neighbours and Neighbourhoods on Major Depressive Disorders in Adults. *Journal of Urban Health*. https://doi.org/10.1007/s11524-025-01055-x

Tutzer, F., Schurr, T., Frajo-Apor, B., Pardeller, S., Plattner, B., Schmit, A., Conca, A., Fronthaler, M., Haring, C., Holzner, B., Huber, M., Marksteiner, J., Miller, C., Perwanger, V., Pycha, R., Schmidt, M., Sperner-Unterweger, B., & Hofer, A. (2024). Relevance of spirituality and perceived social support to mental health of people with pre-existing mental health disorders during the COVID-19 pandemic: a longitudinal investigation. *Social Psychiatry and Psychiatric Epidemiology*, *59*(8), 1437–1448. https://doi.org/10.1007/s00127-023-02590-1

Upenieks, L., Kent, B. V., Nagaswami, M., Gu, Y., Kanaya, A. M., & Shields, A. E. (2024). Do Religion and Spirituality Buffer the Effect of Childhood Trauma on Depressive Symptoms? Examination of a South Asian Cohort from the USA. *Journal of Religion and Health*, *63*(4), 2998–3026. https://doi.org/10.1007/s10943-024-02040-5

Upenieks, L., & Thomas, P. A. (2021). Gaining Faith, Losing Faith: How Education Shapes the Relationship between Religious Transitions and Later Depression. *Journal of Health and Social Behavior*, *62*(4), 582–598. https://doi.org/10.1177/00221465211046356

VanBronkhorst, S. B., Abraham, E., Dambreville, R., Ramos-Olazagasti, M. A., Wall, M., Saunders, D. C., Monk, C., Alegría, M., Canino, G. J., Bird, H., & Duarte, C. S. (2024). Sociocultural Risk and Resilience in the Context of Adverse Childhood Experiences. *JAMA Psychiatry*, *81*(4), 406–413. https://doi.org/10.1001/jamapsychiatry.2023.4900

Wang, C., & Wang, H. (2026). Association between physical activity and mental health (emotional and peer problems) in children and adolescents, a national follow-up study. *PLOS One*, *21*(4), e0345920. https://doi.org/10.1371/journal.pone.0345920

Wang, D., Jiang, Q., Yang, Z., & Choi, J.-K. (2021). The longitudinal influences of adverse childhood experiences and positive childhood experiences at family, school, and neighborhood on adolescent depression and anxiety. *Journal of Affective Disorders*, *292*, 542–551. https://doi.org/10.1016/j.jad.2021.05.108

Wang, Z., Cao, Z., Min, J., Duan, T., & Xu, C. (2025). Associations between device-measured and self-reported physical activity and common mental disorders: Findings from a large-scale prospective cohort study. *BMJ Evidence-Based Medicine*, *30*(1), 45–54. https://doi.org/10.1136/bmjebm-2024-112933

Whittle, S., Simmons, J. G., Dennison, M., Vijayakumar, N., Schwartz, O., Yap, M. B. H., Sheeber, L., & Allen, N. B. (2014). Positive parenting predicts the development of adolescent brain structure: A longitudinal study. *Developmental Cognitive Neuroscience*, *8*, 7–17. https://doi.org/10.1016/j.dcn.2013.10.006

Williams, C. J., Kessler, D., Fernyhough, C., Lewis, G., & Pearson, R. M. (2016). The association between maternal-reported responses to infant crying at 4 weeks and 6 months and offspring depression at 18: a longitudinal study. *Archives of Women’s Mental Health*, *19*(2), 401–408. https://doi.org/10.1007/s00737-015-0592-2

Wolke, D., Zhou, Y., Liu, Y., Eves, R., Mendonça, M., & Twilhaar, E. S. (2025). A systematic review of conceptualizations and statistical methods in longitudinal studies of resilience. *Nature Mental Health*, *3*(9), 1088–1099. https://doi.org/10.1038/s44220-025-00479-3

Wong, S. M. Y., Suen, Y. N., Hui, C. L. M., Lee, E. H. M., Chan, S. K. W., & Chen, E. Y. H. (2026). Connected yet lonely: Prospective influences of loneliness and varying degrees of social connection and isolation on the new onset of major depressive episodes in young people. *Journal of Affective Disorders*, *398*, 120909. https://doi.org/10.1016/j.jad.2025.120909

Wu, M.-H., Chiao, C., & Lin, W.-H. (2024). Adverse childhood experience and persistent insomnia during emerging adulthood: do positive childhood experiences matter? *BMC Public Health*, *24*(1), 287. https://doi.org/10.1186/s12889-024-17774-w

Zhang, L., Fang, J., Zhang, D., Wan, Y., Gong, C., Su, P., Tao, F., & Sun, Y. (2021). Poly-victimization and psychopathological symptoms in adolescence: Examining the potential buffering effect of positive childhood experiences. *Journal of Affective Disorders*, *282*, 1308–1314. https://doi.org/10.1016/j.jad.2021.01.011

Zhou, Z. (2024). Association of Childhood Friendship Experience and Depressive Symptoms Among Older Adults: A Causal Mediation Approach. *The Journals of Gerontology, Series B: Psychological Sciences and Social Sciences*, *79*(4). https://doi.org/10.1093/geronb/gbae019
